# Chemically Fueled Active Transport

**DOI:** 10.1002/anie.202500243

**Published:** 2025-05-28

**Authors:** Christine M. E. Kriebisch, Brigitte A. K. Kriebisch, Gregor Häfner, Héctor Soria‐Carrera, Yanyan Fei, Marcus Müller, Job Boekhoven

**Affiliations:** ^1^ School of Natural Sciences, Department of Chemistry Technical University of Munich Lichtenbergstraße 4 85748 Garching Germany; ^2^ Institute for Theoretical Physics Georg‐August University Friedrich‐Hund‐Platz 1 37077 Göttingen Germany; ^3^ Max Planck School Matter to Life Jahnstraße 29 69120 Heidelberg Germany

**Keywords:** Active transport, Chemical‐fuel‐driven reaction cycle, Molecular pumps

## Abstract

Biology uses chemical potential differences from molecules like ATP to drive membrane pumps, transporting molecules across membranes even against concentration gradients. Here, we report a synthetic system that transports small molecules from an aqueous phase (the sender) to an aqueous receiver across a centimeter‐sized immiscible solvent at the expense of chemical energy. Molecules with high chemical potential (fuels) in the sender phase transiently activate the transporter molecules, which enter the immiscible solvent and exit on the receiver phase side, thus allowing transport from sender to receiver against a concentration gradient. Importantly, we show this active transport mechanism transports molecules across a hydrophobic barrier without complex pumping machinery. Using a reaction‐diffusion model, we identify the critical parameters that determine the efficiency of active transport. Selective transport enables the sorting of a mixture of different molecules. In future work, we will use these design criteria to actively transport molecules across membranes, e.g., of vesicles, at the expense of chemical fuel, thereby mimicking biological processes and enabling the feeding of synthetic cells.

## Graphical abstract

We report a synthetic system that uses chemical potential differences similar to biological systems to transport small molecules across a hydrophobic barrier against a concentration gradient. Instead of a complex pumping machinery, transport happens through the transient activation of transporter molecules. Our reaction‐diffusion model identified key parameters for efficient transport, enabling selective sorting of molecular mixtures.

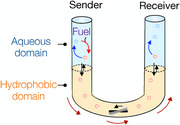

## Introduction

**Scheme 1:**
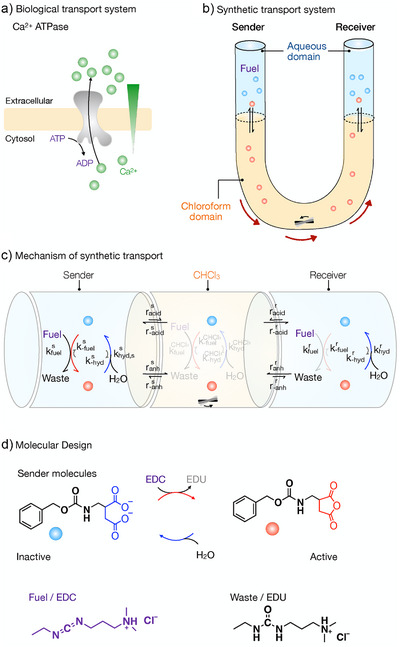
Biological and synthetic active transport systems. a) Biological active transport of Ca^2+^ ions through the cell membrane. At the expense of the biological fuel ATP, the Ca^2+^ ATPase pump pumps Ca^2+^ ions from the cell inside, i.e., the sender, to the cell outside, i.e., the receiver, via the cell membrane against the Ca^2+^ ion gradient. b) Synthetic active transport system of a dicarboxylate sender molecule through a chloroform domain. At the expense of the chemical fuel EDC, the dicarboxylate activates and forms the anhydride product in the sender. The anhydride product partitions in the chloroform and is transported from the sender to the receiver. At the receiver, the anhydride hydrolyzes to the dicarboxylate sender molecule. c) Mechanism of chemically fueled active transport. The sender (S) and receiver (R) are separated by a chloroform phase. Fuel activates the sender molecule Cbz‐D (blue ball), converting it to the anhydride (anh, red ball). Anhydride and acid Cbz‐D partition into the chloroform, cross it, and leave it at the receiver side. The anhydride partitioning is higher compared to the acid Cbz‐D. At the receiver side, anhydride deactivates via hydrolysis (hyd). There is a spatial asymmetry of fuel concentration in the sender and receiver phase, as well as a difference in $r_{\mathrm{Ac}}^{R/S}$ and $r_{\mathrm{An}}^{R/S}$, which is achieved by the difference in hydrophilicity. This creates a kinetic asymmetry, leading to active transport of the sender molecule acid Cbz‐D. d) Molecular structure of sender molecule Cbz‐D, Cbz‐D anhydride, fuel EDC (1‐ethyl‐3‐dimethylaminopropyl carbodiimide), and waste EDU (1‐[3‐(dimethylamino)propyl]‐3‐ethylurea).

Biology uses pumps to maintain concentration gradients,^[^ ^1^, ^2^ ^]^ regulate blood pressure,^[^ ^3^ ^]^ cell volume,^[^ ^4^ ^]^ or electrolyte balance.^[^ ^5^ ^]^ For instance, the Ca^2+^ ATPase is a pump that transports molecules from the cytosol to the extracellular medium against a concentration gradient, using ATP in the process (Scheme 1a). Specifically, ATP phosphorylates the ATPase, causing a conformational change that triggers the transport of Ca^2+^ ions.^[^ ^6^ ^]^ Dephosphorylation of the pump ensures it continuously operates. This sophisticated mechanism inspires the creation of supramolecular pumps to transport molecules directionally across barriers with potential applications in nanotechnology. Examples are pumps that purify water from pollutants^[^ ^7^, ^8^ ^]^ or aid in biomedical applications. The field has mainly focused on molecular pumps^[^ ^9^, ^10^, ^11^ ^]^ based on interlocked systems,^[^ ^12^ ^]^ i.e., systems that pump a noncovalently bound guest from one site to another.^[^ ^13^, ^14^ ^]^ For example, a pump that transports a macromolecular ring along a polymer backbone powered by chemical fuel was described.^[^ ^11^, ^13^, ^15^, ^16^ ^]^ However, there has been a lack of active molecular transport of small molecules and ions across membranes.^[^ ^8^, ^17^ ^]^ So far, systems have shown passive transport that allows for the diffusion of ions down a concentration gradient (equilibration) across membranes or liquid barriers.^[^ ^18^, ^19^, ^20^, ^21^, ^22^, ^23^ ^]^ Notably, earlier studies reported molecular transport, where photoswitchable chemical hosts shuttle ions or molecules from a sender to a receiver phase driven by light^[^ ^8^, ^24^, ^25^ ^]^ or electrochemistry^[^ ^26^, ^27^ ^]^ and the generation of photocurrent based on a light‐driven proton pump in an artificial liquid membrane.^[^ ^25^ ^]^ A similar synthetic design driven by the chemical potential of fuels remains a challenge.

In this work, we demonstrate the transport of molecules from an aqueous sender phase to an aqueous receiver phase across a centimeter‐sized chloroform domain (Scheme 1b). The transport occurs directionally and against a concentration gradient from the sender to the receiver phase via a chloroform barrier by transducing high‐chemical‐potential fuel in the sender phase into a waste molecule with low chemical potential. Specifically, a molecule reacts with the fuel to become chemically activated (red ball in Scheme 1c), which enables it to partition in the chloroform barrier. If it escapes to the receiver phase and deactivates, where it is hindered from being transported back. Thus, molecules pass the chloroform barrier directionally. Notably, we achieve this active and directed transport without complex machinery but simply by transiently changing the partitioning coefficient of the transporter molecule by a carbodiimide‐driven chemical reaction cycle.^[^ ^28^, ^29^, ^30^ ^]^ The key, therefore, is the difference in fuel concentration in the sender and receiver phases that change the activation rate, thus introducing kinetic asymmetry and diffusion asymmetry to the reaction network and ultimately driving active transport. This experiment illustrates Maxwell's Demon thought experiment, in which molecules are sorted by a hypothetical demon based on their velocity.^[^ ^24^, ^31^, ^32^, ^33^, ^34^, ^35^, ^36^ ^]^ In our experiment, our hypothetical demon selects molecules based on their activation state (Scheme 1c). The selection occurs selectively in the sender phase because of the “information” encoded in the addition of fuel to the sender phase only.^[^ ^37^ ^]^ This chemical conversion lowers the kinetic barrier to transport from the sender phase without affecting the barrier for transport from the receiver phase. The selection based on activation state is a consequence of this chemically fueled event. Thus, the proposed mechanism for active transport bears several hallmarks of an information ratchet.^[^ ^14^, ^37^, ^38^ ^]^ Our experimental work, combined with a numerical reaction‐diffusion model, provides guidelines for using chemically‐fueled reaction cycles to regulate the active transport of molecules across hydrophobic domains. Besides, the group of Ragazzon reports a similar system that elaborates more on the active transport of molecules.^[^ ^39^ ^]^ Our work specifically focuses on understanding the parameters that influence transport efficiency.

## Results and Discussion

### Experimental Setup

Our synthetic active transport system uses a glass U‐tube in which a chloroform phase separates an aqueous sender from an aqueous receiver phase (Figure S1A). The sender contains 200 mM MES buffered water at pH 6 and a dicarboxylate as a transporter molecule. The receiver also contains aqueous buffered at pH 6 by 200 mM MES to avoid effects from significant pH differences (Scheme 1b).^[^ ^18^ ^]^ By stirring the chloroform phase but not the aqueous phases, we ensure a homogeneous mixing of the chloroform phase. In our design, the transporter molecule and fuel partition poorly in the chloroform phase to limit passive transport. Passive transport refers to molecules diffusing from one side to the other without fuel, negating the active transport's work. We used the water‐soluble charged EDC (1‐ethyl‐3‐dimethylaminopropyl carbodiimide) as fuel and the anionic‐charged dicarboxylate, Cbz‐D, as a transporter molecule to meet this requirement (Scheme 1d).^[^ ^40^, ^41^ ^]^ The application of fuel activates the poorly partitioning transporter molecule via the extremely short‐lived O‐acylisourea intermediate that we cannot detect^[^ ^30^, ^42^ ^]^ in its corresponding anhydride, which is expected to partition better due to charge neutrality. Hence, if an activated transporter molecule diffuses to the sender–chloroform interface, it may enter the chloroform phase, which hydrolyzes only at a vanishingly low rate. No appreciable anhydride hydrolysis was observed in wet chloroform that has been saturated with the aqueous buffer (Figure S1B,C). Thus, $k_{\mathrm{hyd}}^{C}$ is negligible, and the reaction network in Scheme 1c reduces.^[^ ^43^ ^]^ The stirring ensures the chloroform phase is well mixed, and the activated molecule can also exit the chloroform phase on the receiver side, where it hydrolyzes and remains trapped.

**Figure 1:**
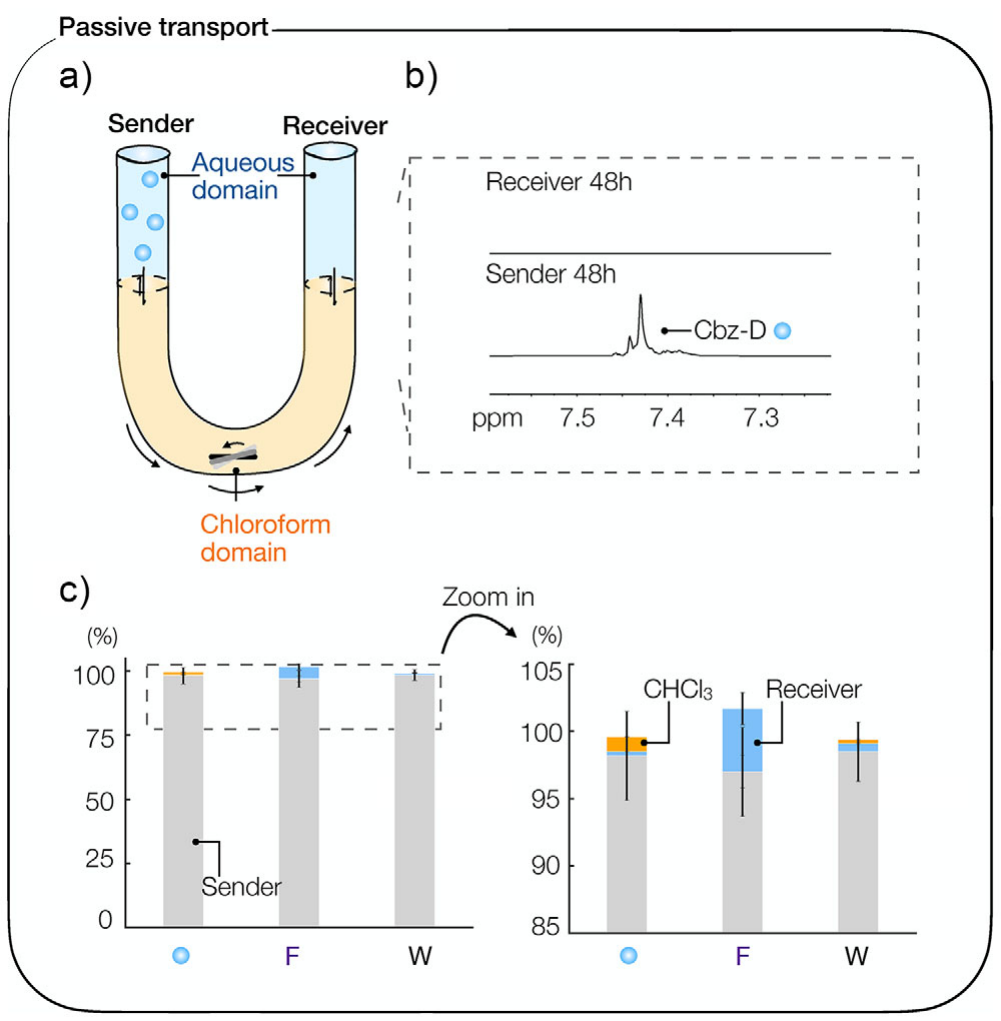
Passive transport in the synthetic transport system. a) Schematic overview of the synthetic transport system. A U‐shaped glass tube (*⌀* = 5 mm) filled with 500 µL CHCl_3_ (chloroform phase) and 600 µL 200 mM MES, pH 6 sender and receiver. The sender contains either 10 mM Cbz‐D (sender molecule), 100 mM EDC (fuel), or 100 mM EDU (waste). A stirring bar stirs the chloroform phase at 1000 rpm. b) ^1^H NMR spectrum of the sender and receiver after 48 h shows that no Cbz‐D is present in the receiver phase. c) Quantitative ^1^H NMR analysis of the sender and receiver after 48 h of the Cbz‐D, EDC (F  =  fuel), and EDU (W  =  waste). Error bars represent the standard deviation of the mean (*n* = 3). The percentage refers to the starting concentration of acid.

We measured the passive transport of 10 mM Cbz‐D, 100 mM EDC, and 100 mM EDU (1‐[3‐(dimethylamino)propyl]‐3‐ethylurea) from the sender to the receiver in three U‐tube setups. We report our results in the percentage of the molecules in the sender phase transported (%). Uncertainties are given as standard deviations of the mean of a triplicate if not otherwise indicated. Using ^1^H NMR spectroscopy, we measured the amount of Cbz‐D, EDC, and EDU in the sender and receiver after 48 h (Figure 1a). We found that only 0.3 ± 0.01% of Cbz‐D, 5 ± 1.2% EDC, and 0.6 ± 0.03% EDU passively diffused from the sender to the receiver within two days (Figures 1b,c and S2). Around 1.4 ± 0.03% of Cbz‐D, 0 ± 0.01% of EDC, and 0.9 ± 0.03% of EDU resided in the chloroform phase. In conclusion, these findings demonstrate that the direct passive diffusion of acid molecules across the chloroform phase without reactions is extremely slow.

Next, we tested if EDC can convert the transporter molecule, Cbz‐D, into its anhydride state. In a vial, we fueled 10 mM Cbz‐D with 100 mM EDC in 200 mM MES pH 6 and monitored the formation of the Cbz‐D‐anhydride in response to EDC by high‐performance liquid chromatography (HPLC, Figure S3 and Table S1). We used a previously developed benzylamine quench assay to increase the accuracy.^[^ ^44^ ^]^ To describe the reaction kinetics of the fuel conversion, activation, and deactivation, we assume all reactions are irreversible and follow mass‐action kinetics, resulting in a set of ordinary differential equations.^[^ ^45^, ^46^ ^]^ These kinetics are derived from a thermodynamically consistent formulation and properly justified in the Supporting Notes. We used the time evolutions of concentrations in a homogeneous system to fit the kinetic coefficients in the model using least‐square fitting (see Supporting Notes for more details). We found that the anhydride half‐life is 42 s (Figures S3–S5; Tables S2 and S3), which corresponds to *k*_hyd_ = 2 min^−1^. Finally, we found no evidence of assembly before and after fueling by turbidity measurements (Figure S6).

### Chemically Fueled Transport

**Figure 2:**
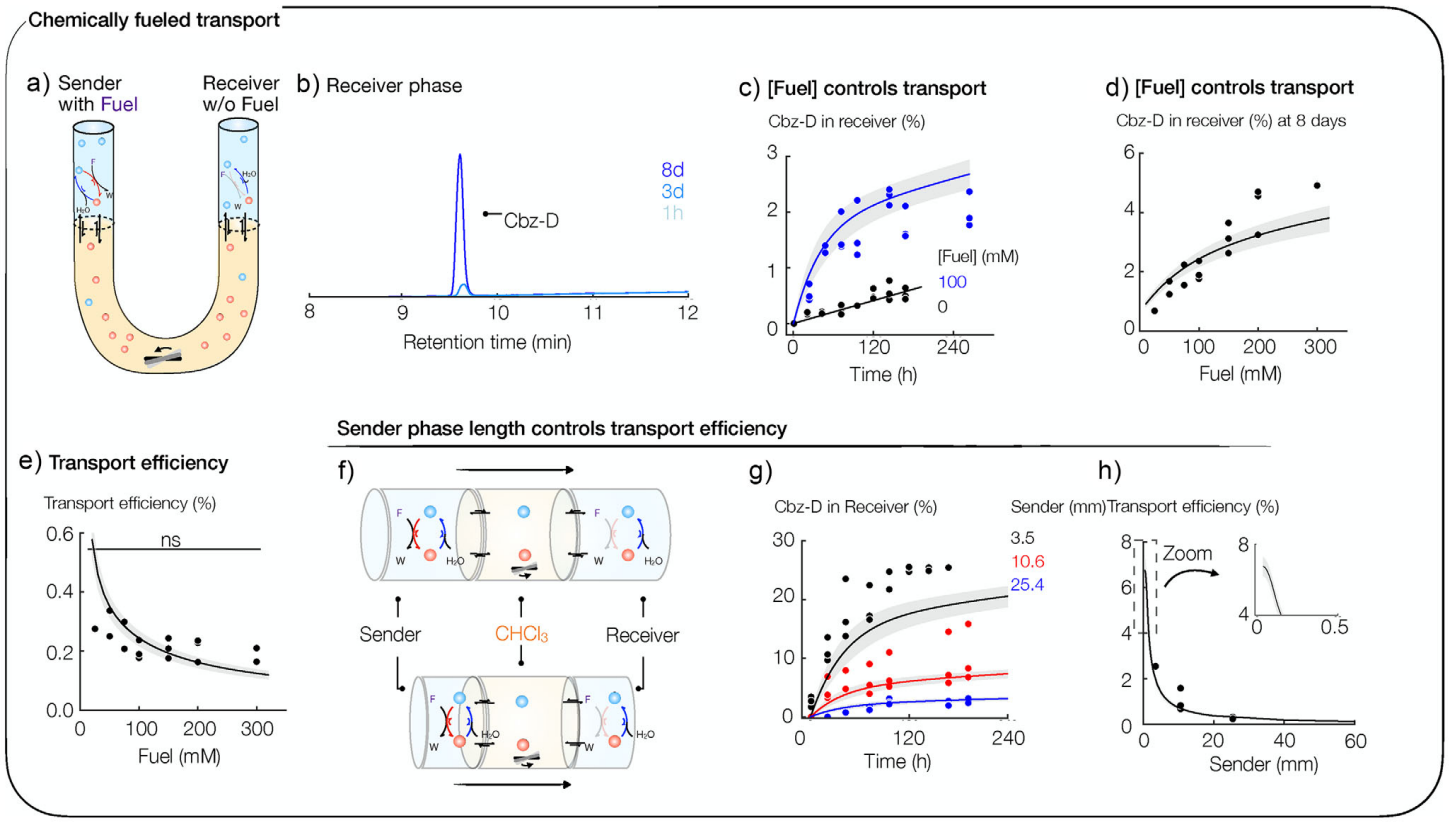
Chemically fueled transport in the synthetic transport system. a) Schematic overview of the synthetic transport system. A U‐shaped glass tube (*⌀* = 5 mm) filled with 500 µL CHCl_3_ (chloroform phase) and 600 µL 200 mM MES pH6 sender and receiver. The sender contains 10 mM Cbz‐D (transporter molecule) and 100 mM EDC (fuel) or 10 mM Cbz‐D and 100 mM EDU (waste). A stirring bar stirs the chloroform phase at 1000 rpm. b) Representative HPLC chromatogram of Cbz‐D on the receiver after 1 h, 3 days, and 8 days. c) Percentage of Cbz‐D transported to the receiver as a function of time in the presence of 0 mM and 100 mM EDC fuel. d) Percentage of Cbz‐D transported to the receiver as a function of initial fuel concentration, [F]^S^(*t*  =  0). e) Efficiency of Cbz‐D transport as a function of initial fuel concentration, [F]^S^(*t*  =  0). f–h) Influence of the sender length on the chemically fueled transport. f) Scheme represents the change of the experimental conditions from long to shorter sender phase lengths. The sender phase length impacts the transport efficiency, as shown in (g) and (h). g) Transport of Cbz‐D% over time. h) Transport efficiency of Cbz‐D as a function of sender length. We used a least‐square method to fit the data for all three datasets (sender length = 0.35 cm, 1.06 cm, 2.54 cm). From this, we obtained the model parameters that fit, on average, all three datasets best. We used those model parameters to predict the experimental data on the evolution of transporter molecules for various initial fuel concentrations (Figure 2c–e). Interestingly, the transport efficiency increases significantly when the fuel concentration goes to zero (Figure 2h), caused by passive transport, i.e., the diffusion of nonactivated Cbz‐D acid molecules through the chloroform phase, which does not require EDC. We hypothesize that this characteristic of the system causes the reaction‐diffusion model to underestimate the experimental data for smaller sender lengths, as seen in Figure 2h. We expect the larger variability between the replicates of the shortest versus longest sender length to be found in the nature of the experimental setup. Although a large sender length allows an easy taking of aliquots, it becomes more difficult with a decrease in the sender length. (c–e, g, and h) All experiments were carried out in triplicate. The curves represent data from our reaction‐diffusion model. c), d), and g) Percentage refers to the starting concentration of acid. e) Statistical analysis was performed using a one‐way Welch‐ANOVA test: ns, not significant (*p* > 0.05). e) and h) Based on the amount of Cbz‐D transported and the initial fuel concentration, we calculated the transport efficiency after 8 days as $\frac{{\left[\mathrm{Ac}\right]}^{R}\left(t=8d\right)}{{\left[F\right]}^{S}\left(t=0\right)}$.

Before testing for chemically fueled transport against a concentration gradient (vide infra), we determined whether the transporter molecule transports from the sender to the receiver after being activated to its corresponding anhydride by chemical fuel. Thus, we added 100 mM EDC to 10 mM Cbz‐D in the sender phase (Figure 2a). Over 8 days, we took daily aliquots from the sender and receiver phases and measured the amount of EDC and Cbz‐D using high‐performance liquid chromatography (HPLC, Figure 2b). We found that, within an hour, all EDC was consumed, and 5.0 ± 1.2% Cbz‐D partitioned into the chloroform phase (Figures 2c and S7A). Over 5 days, 2% ± 0.3% Cbz‐D transported to the receiver phase, whereas 97 ± 1.3% Cbz‐D remained in the sender (Figure 2c). Hence, the majority of Cbz‐D molecules that traveled to the receiver side, did so by reacting with EDC in the sender phase, entering the chloroform phase as the acid and exiting on the receiver side, rather than directly passing the chloroform phase as anhydride without reactions. Moreover, when monitoring the fuel, we found only a small amount of EDC or EDU (0.5 ± 0.1%) is transported to the receiver side on the timescale of the experiment (Figure S7B). This result implies a fourfold greater active transport than nonfueled passive transport, i.e., without fueling, around 0.5% of Cbz‐D were transported from sender to receiver after 5 days (Figure 2c). As more fuel implies more anhydride formation, we hypothesized it should control the amount of transported Cbz‐D. Indeed, we found that by fueling with various fuel concentrations, we can regulate the amount of transported Cbz‐D between 0.64% ± 0.05 and 5.4% ± 0.8 (Figure 2d). Notably, the unwanted side product *N*‐acylisourea by rearrangement of *O*‐acylisourea was not observed in the reaction cycle, similar to previous studies (Figure S8).^[^ ^47^, ^48^, ^49^, ^50^ ^]^

Based on the amount of Cbz‐D acid transported, [Ac]^R^(*t*) − [Ac]^R^(*t* = 0), and the initial fuel concentration [F]^S^(*t* = 0), we calculated the transport efficiency using:

$$\eta \;\left(t\right)=\frac{{\left[\mathrm{Ac}\right]}^{R}\left(t\right)-{\left[\mathrm{Ac}\right]}^{R}\left(0\right)}{{\left[F\right]}^{S}\left(0\right)}$$

Note that this definition of efficiency only quantifies the number of fuel molecules required for transport but not the (free‐)energy cost. In the majority of the setups of this work, the initial acid concentration in the receiver is zero, simplifying the equation. An efficiency of 100% implies each molecule of EDC transported one transporter molecule to the sender phase. We found *η* = 0.25% ± 0.05% when using 100 mM EDC (Figure 2e). Disappointingly, this efficiency implies that 400 molecules of EDC are needed to transport one molecule of Cbz‐D from sender to receiver. With an increasing amount of initial fuel, the total amount of Cbz‐D molecules transported increased, but the transport efficiency did not change significantly.

### Regulating the Fueled Transport

We hypothesize that the successful transport proceeds in four critical steps: 1) a transporter molecule is activated in the sender phase, 2) it diffuses to the interface with chloroform and partitions into it, 3) it diffuses and is advected by the stirring in the chloroform phase, from where it may partition into the receiver phase, and 4) it is deactivated on the receiver side and can no longer escape. At each step, efficiency‐decreasing processes occur. For example, in step 1, the activation of the transport molecule occurs via an intermediate *O*‐acylisourea, which can decay before converting to the activated transport molecule—the anhydride. Similarly, the fuel itself can decay via hydration. In step 2, activated transport molecules (away from the sender–chloroform interface may) likely deactivate before reaching the chloroform phase. In step 3, activated transport molecules can also repartition into the sender phase. In step 4, activated transport molecules can re‐enter the chloroform phase before deactivating. Moreover, molecules can diffuse back through passive transport. To optimize the transport efficiency, we studied the different parameters that lead to an efficiency loss at steps 1, 2, 3, and 4.

In step 1, the loss of activated molecules via the *O*‐acylisourea hydrolysis and the loss of fuel via its hydration decreases the efficiency. The least‐square fitting of the concentration profiles indicated that neither of these two pathways played a significant role (Figures S3–S5). In both steps 2 and 3, transport of molecules through chloroform is essential. We, thus, determined if this is limited by diffusion within the chloroform by testing different stirring speeds, interface cross‐sections, and lengths of the chloroform phase. First, we tested the influence of the stirring speed. We found that increasing the stirring from 1000 to 1600 rpm does not significantly influence the transport efficiency (Figure S9A–C), indicating the chloroform phase is well mixed. It also demonstrates that hydrolysis in the chloroform does not play a major role, i.e., a faster transfer from sender to receiver by faster mixing did not result in the loss of transported molecules, affecting the efficiency.

We found that increasing the interface area from 79 to 113 mm^2^ did not significantly influence the transport efficiency (Figure S9D–F). Furthermore, we found that increasing the length of the chloroform phase from 25 to 35 mm does not significantly modify the transport efficiency (Figure S9G–I), corroborating our finding that the chloroform phase is well mixed. Notably, our experimental findings on the passive transport, presented, for example, in Figure 2c, show that passive diffusion of the acid is a slow process, contributing little to the overall transport. We conclude that step 4, “back” diffusion from receiver to sender phase, can thus be considered insignificant.

To further understand the details and efficiencies of the transport in each step, we devised a simple 1D reaction‐diffusion model that describes the time‐dependent transport of Cbz‐D across the hydrophobic barrier in response to the fuel (see Supporting Notes for more details). The reaction‐diffusion model considers the fuel‐driven activation and spontaneous hydrolysis of activated molecules and the partitioning of the activated molecules inside the hydrophobic domain (chloroform). It describes the diffusion of molecules in the sender and receiver phases while conceiving the chloroform phase as homogeneously mixed through stirring. Hence, there is a finite rate at which the interfaces equilibrate, determining how many molecules cross the sender–chloroform and chloroform–receiver interfaces. The time‐ and space‐dependent concentration profiles are described by [*I*]^*X*^(*x*,*t*) where *I*  =  Ac,   An,  F, denotes acid, anhydride and EDC fuel, respectively. *X*  =  S,  R denotes the sender and receiver phase. The concentrations’ time evolution follows a diffusion equation with sinks and sources stemming from reactions and the interface equilibration:

$$\frac{\partial {\left[I\right]}^{X}\left(x,t\right)}{\partial t}=D\frac{\partial^{2}{\left[I\right]}^{X}\left(x,t\right)}{\partial x^{2}}-s_{I}^{X}\left(x,t\right)+r_{I}^{X}\delta \left(x-x_{\mathrm{XC}}\right).$$

On the right‐hand side, the first term stems from the diffusion with the diffusion coefficient, *D*. The second term denotes local changes in concentrations due to reactions, which have the same form as the above‐described reaction model. The third term leads to interface equilibration, where δ is the Dirac delta function and *x*_SC_ and *x*_RC_ denote the two interface positions. The concentrations in the chloroform phase, [*I*]^*X*^, which has length *L_C_*, have no spatial dependence, and their time evolution simplifies to:

$$\frac{d{\left[I\right]}^{C}\left(t\right)}{dt}=s_{I}^{C}\;\left(t\right)-\frac{1}{L_{C}}\left(r_{I}^{s}\left(t\right)+r_{I}^{r}\left(t\right)\right).$$

The molecules interact differently with the aqueous solution than with the chloroform. This interaction difference is captured in the model by an excess chemical potential Δμ_*I*_ for each species. In equilibrium, this leads to a discontinuity of the concentrations at the two interfaces, i.e., a difference in partitioning into the two phases. To approach this equilibrium condition, the interface‐equilibration term takes the form:

$${\;r}_{I}^{X}=-r\frac{{\left[I\right]}^{X}\left(x_{XC}\right)-e^{-\beta \Delta \mu_{I}}{\left[I\right]}^{C}}{e^{-\beta \Delta \mu_{I}}+1}$$

for β  = (*k*_B_ *T*)^−1^  with the Boltzmann constant *k*_B_ and temperature *T*. The proportionality factor, *r*, determines the rate at which interface equilibration occurs, and equilibrium is reached when the numerator vanishes. Whether molecules enter the chloroform phase is thus determined by two parameters, the rate constant, *r*, and the excess chemical potential, Δμ_*I*_, which implicitly determines the partitioning into the chloroform phase. Model parameters not obtained by experimental measurements—the diffusion constants, the rate of transfer across the interfaces, and the excess chemical‐potential differences of acid and anhydride—were determined using least‐square fitting of the experimental data from Figure 2c,g. We used the obtained model parameters to predict the experimental data on the evolution of transporter molecules for various initial fuel concentrations (Figure 2c–e). The transport efficiency increases significantly when the fuel concentration approaches zero (Figure 2e), which is caused by passive transport, i.e., the diffusion of nonactivated Cbz‐D molecules through the chloroform phase, which does not require EDC. This is, of course, the side effect of vanishing initial Cbz‐D concentration in the receiver phase. When starting with equal initial Cbz‐D concentration in both phases, where the passive transport counteracts the active one, the efficiency is small for low initial fuel concentrations.

Using the reaction‐diffusion model, we investigated how step 2, i.e., the activation in the sender phase and its diffusion to the chloroform phase, is involved in transport efficiency. We found minimizing the length of the aqueous sender phase is crucial. As the sender length increases (the distance), activated molecules must diffuse to the sender–chloroform interface, which can exceed their lifetime *t*—the time needed for 1/e molecules to decay. In other words, only a small fraction of the volume close to the chloroform interface in the sender phase contributes to the transport. This volume is on the order of $\sqrt{Dt}\approx$ 1 mm. In the region farther away than 1 mm, the concentration of activated sender molecules is homogeneous, which means that molecules are activated and deactivated but not transported to the receiver phase (see Supporting Notes and Figure S10). We reduced the sender length from 25.4 to 3.5 mm in our experiments. This reduction increased the efficiency by 10‐fold to 2.5 ± 0.01%, in line with the reaction‐diffusion model (Figure 2f–h). Thus, only 40 fuel molecules are needed to transport one Cbz‐D molecule to the receiver. Moreover, the reaction‐diffusion model showed that a further decrease in the sender phase length increases efficiency up to a value of approximately 7%. Notice that the maximally achievable efficiency is [Ac]^S^(*t*  =  0)/[F]^S^ (*t*  =  0) =  10% for the given ratio of initial Cbz‐D and EDC‐fuel concentration. Although this was experimentally not feasible, the reaction‐diffusion model is particularly powerful as it offers an optimistic prospect for future miniaturization, such as using microfluidics or phospholipid membranes.

### Selective Transport

**Figure 3:**
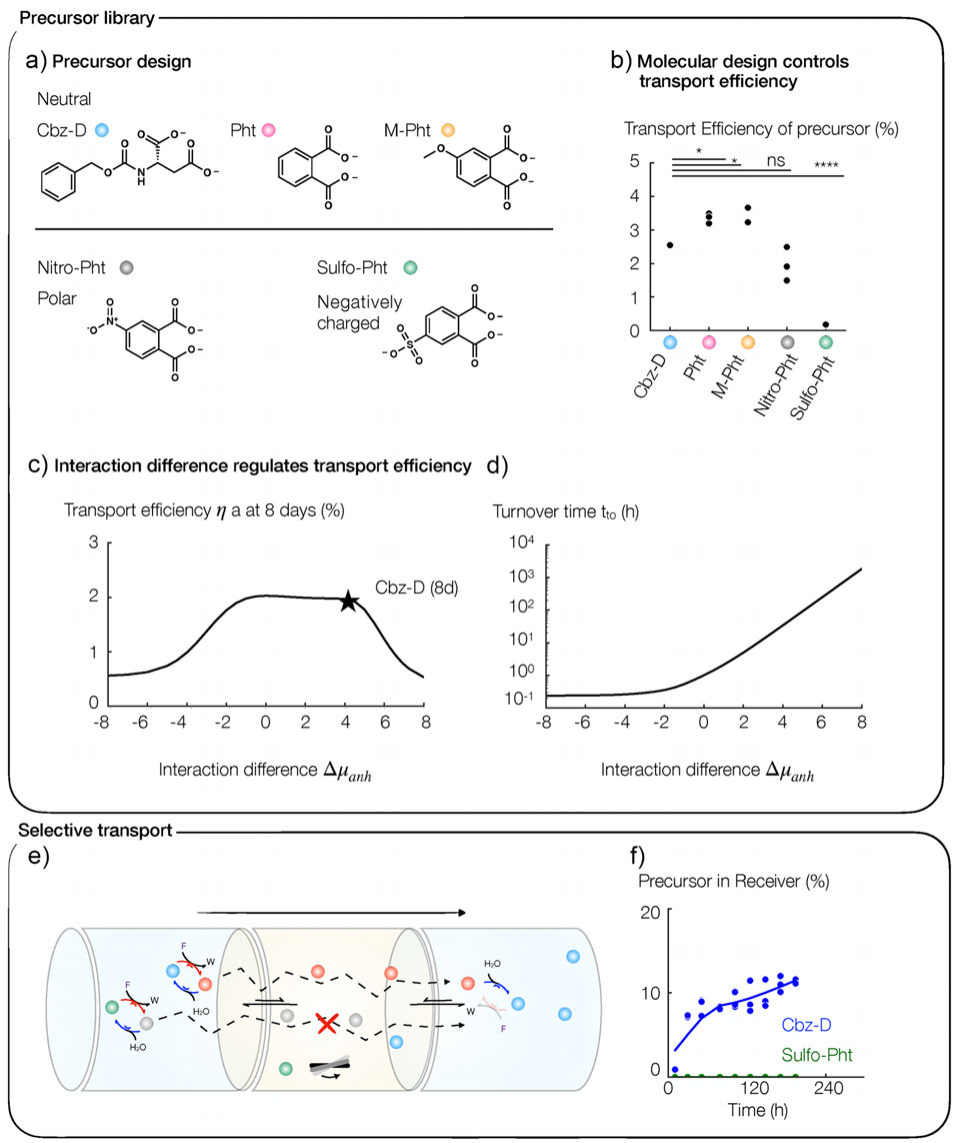
Selective transport of molecules. a) Molecular design of sender molecules. b) The efficiency of Cbz‐D, phthalic acid, methoxy‐phthalic acid, nitro‐phthalic acid, and sulfo‐phthalic acid transport after fueling with 100 mM EDC after 7 days or in case of methoxy‐phthalic acid with 5 mM EDC after 10 days. Based on the amount of acid transported, we calculated the transport efficiency using η(*t*)  = [Ac]^R^ (*t*)/[F]^S^(*t*  =  0). Efficiency at c) eight days and d) turnover time as a function of the interaction difference of Cbz‐D anhydride with the chloroform phase. The star indicates the reaction‐diffusion model result for Cbz‐D as the sender molecule. e) Schematic illustration of selective transport of molecules across a hydrophobic barrier. f) Percentage of Cbz‐D and sulfo‐phthalic acid transported from sender to receiver when fueling a 1:1 mixture of Cbz‐D and sulfo‐phthalic acid with 100 mM EDC. The percentage refers to the starting concentration of acid. Lines are guides for the eye.

We further investigated the efficiency loss related to step 2, i.e., the step in which the freshly activated molecule partitions into the chloroform phase, and step 3, i.e., the step in which it leaves the chloroform again. We tested several transporter molecules differing in their half‐life, partitioning coefficient, and their interface‐crossing rate. Specifically, next to the charge‐neutral Cbz‐D anhydride, phthalic anhydride, and methoxy‐phthalic anhydride, we tested the polar nitro‐phthalic anhydride and the negatively charged sulfo‐phthalic anhydride (Figure 3a). Again, we found no evidence of assembly before and after fueling when fueling 10 mM Cbz‐D, 10 mM phthalic acid, 10 mM nitro‐phthalic acid, and 10 mM sulfo‐phthalic acid with 100 mM EDC and for 10 mM methoxyphthalic acid with 5 mM EDC by a turbidity measurement, confirming that phthalic acid, methoxy‐, nitro‐ and sulfo‐phthalic acid were well‐dissolved (see Figure S11). Fueling 10 mM methoxy phthalic acid with more than 5 mM EDC results in the formation of self‐assemblies. Phthalic acid, methoxy‐, nitro‐ and sulfo‐phthalic acid successfully crossed the hydrophobic barrier after activation with EDC. However, negatively charged sulfo‐phthalic acid was only transported with a 0.2% efficiency, showing the importance of partitioning in chloroform. In contrast, phthalic anhydride and methoxy‐phthalic anhydride molecules were transported roughly 15 times more effectively, i.e., with ∼3.4% efficiency, and the polar nitro‐phthalic anhydride molecules were transported roughly as effectively as Cbz‐D molecules (Figures 3b and S12–S16). When determining the kinetics, we found that the nitro‐ and sulfo‐phthalic anhydrides have much shorter lifetimes (7.2  and 6 s) compared to Cbz‐D anhydride, phthalic anhydride, and methoxy‐phthalic anhydride that lack the electron‐withdrawing rest (Tables S2 and S3). Due to their longer half‐life, phthalic anhydride and Cbz‐D anhydride have a higher chance of entering the chloroform phase before getting deactivated. Besides, our reaction‐diffusion model shows that the activated phthalic and methoxy phthalic anhydrides show a higher partitioning in the chloroform phase compared to sulfo‐phthalic, nitro‐phthalic anhydrides, and Cbz‐D anhydride. Thus, a combination of longer half‐life and better partitioning due to molecular design results in a 1.3 times higher transport efficiency of phthalic acid and methoxy‐phthalic acid compared to Cbz‐D.

We used the reaction‐diffusion model to understand the role of the partitioning coefficient and interface crossing in the transport. The transfer rate across the interface quantifies the kinetics at which equilibration occurs at the interface. The efficiency of the transportation process is directly proportional to this rate. As a measure for the partitioning coefficient, we used the excess chemical‐potential difference of the anhydride, Δµ_An_, which describes the difference in interaction of the anhydride with water and chloroform. It is negative if molecules interact preferentially with water, whereas it is positive if the opposite is true. Partitioning at the interface leads to a higher local concentration in the more attractive phase. In the following, we refer to this quantity simply as the interaction difference. Figure 3c depicts the transport efficiency after 8 days as a function of the interaction difference, exemplary for the reaction kinetics of Cbz‐D. The transport efficiency decreases if it is unfavorable for molecules to enter the chloroform, represented by an interaction difference of −8 *k*_B_ *T*. Conversely, with a high attractive interaction difference of +8 *k*_B_ *T*, molecules remain trapped in the chloroform, hindering efficiency after 8 days. The ideal scenario is in‐between, ensuring effective transport by avoiding extremes hindering transfer. The same qualitative dependence can be found with the reaction kinetics of the other molecules. In the reaction‐diffusion model, sulfo‐phthalic acid partitions weaker into the chloroform at a drastically reduced interface‐crossing rate, potentially due to its permanent negative charge, and transports with low efficiency. The combined dataset thus shows that only molecules can be transported if they can be converted to an apolar species.

Notably, the transport efficiency is time‐dependent as it is linked to the turnover time (Figure 3d), i.e., the time it takes for 1/e of the transported molecules to enter the receiver phase. The turnover time is small for negative interaction differences because molecules either cannot enter the chloroform phase or leave it immediately. In this regime, the turnover time is approximately constant as the transport is limited by partitioning. For neutral and positive interaction differences, the transport efficiency increases exponentially with the interaction difference as the anhydride molecules prefer to stay in the chloroform, resulting in small transfer rates across the interface (Figure 3d). For example, when monitoring transport efficiency as a function of interaction difference after 12 h or 2 days instead of 8 days, the transport efficiency decreases already for an interaction difference of +3 *k*_B_ *T*, whereas after 8 days, this decrease is observed only above +6 *k*_B_ *T* (Figure S17). Put differently, the partitioning of molecules in the chloroform phase, determined by the interaction difference, is critical, particularly with longer experiment times.

The combined data set suggests that sulfo‐phthalic acid has lower transport efficiency across the chloroform barrier because it does not partition well, has a small transport rate across the interfaces, and has a short half‐life. Additionally, the similar transport efficiency of nitro‐phthalic acid and Cbz‐D and the roughly 0.7% higher transport efficiency of phthalic acid compared to nitro‐phthalic acid suggests that partitioning and half‐life are important for transport efficiency. Thus, by increasing the half‐life due to a decreased anhydride hydrolysis rate and balancing the partitioning, we can, by molecular design, increase the transport efficiency as we decrease the futile cycle of anhydride hydrolysis without entering the chloroform phase.

The relation between partitioning and transport efficiency enables the simple sorting of molecules based on their partitioning into the hydrophobic phase after activation. We designed an experiment where we mixed sulfo‐phthalic acid and Cbz‐D. Although the fuel chemically activates both, only Cbz‐D was selectively transported to the receiver upon fueling this mixture, whereas sulfo‐phthalic acid remained in the sender (Figure 3e,f).

### Chemically Fueled Transport Against a Concentration Gradient

**Figure 4:**
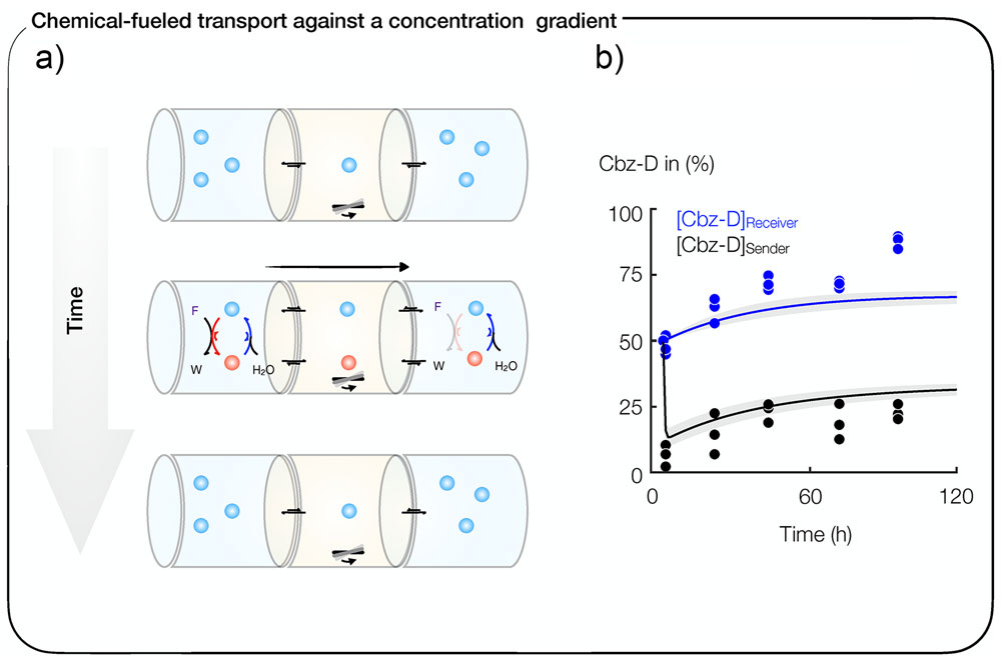
Chemically fueled active transport. a) Schematic illustration of chemically fueled active transport. Molecules transport from sender to receiver against a concentration gradient. b) Percentage of Cbz‐D in sender and receiver after adding 100 mM fuel to the sender. Lines show the results from the reaction‐diffusion model, with fit parameters taken from the data in Figure 2c,h. Experiments are done in triplicate. The percentage refers to the starting concentration of acid.

Finally, we wondered whether we could actively transport molecules against their concentration gradient. To do so, we conducted an experiment in which Cbz‐D was added to both the sender and receiver at a concentration of 8 mM (Figure 4a). After fueling the sender with 100 mM of EDC, we observed a 20%–30% increase in Cbz‐D in the receiver over 8 days, whereas the sender initially decreased by 50% but gradually recovered to 70%–80% over 8 days (Figure 4b). Notably, we could reproduce the data of both experiments with our reaction‐diffusion model to a good degree based on the previously determined parameters.

Finally, we demonstrated that chemically fueled transport can also occur across the hydrophobic barrier dichloromethane (Figure S20). Notably, the passive transport of Cbz‐D across dichloromethane was threefold lower than chloroform, 1% compared to 3% after 3 days, respectively (Figure S20). This shows that dichloromethane, which is significantly less hygroscopic than chloroform, represents a more ideal “nonaqueous” barrier that would minimize passive diffusion of the diacid and hydrolysis of the anhydride during transport.

## Conclusion

Our study demonstrates the chemically fueled active transport of small molecules from a sender to a receiver across a centimeter‐sized chloroform or dichloromethane barrier. Notably, the activated molecule acts as the transporter, eliminating the need for external pumps, unlike biological examples such as the Ca^2+^‐pump. However, as deduced from our reaction‐diffusion model, this transport only works efficiently if the small molecules are activated close to the phase boundary, have a long lifetime, rapidly transfer across the interface, and efficiently partition between aqueous and chloroform domains. Otherwise, transport becomes inefficient. To eliminate these constraints, nature might, in addition to other reasons, have evolved its more complex pump machinery.

By adjusting the amount of chemical fuel, we control the yield of transporter molecules through repetitive fuel addition. Varying the interface‐to‐aqueous ratio—achieved by shortening the sender length—allows us to regulate transport efficiency. Moreover, we showed that we can selectively transport molecules, which allowed us to sort a mixture of two different types of molecules in a scenario illustrating Maxwell's Demon thought experiment.^[^ ^24^, ^31^, ^32^, ^33^, ^34^, ^35^, ^36^ ^]^

In the future, we aim to apply these design principles to transport molecules across membranes, mimicking biological processes. Our findings of directionally transporting molecules against concentration gradients across barriers have implications for designing new types of cargo transport in medical and material applications.

## Author Contributions

J.B., C.M.E.K., and B.A.K.K. conceived the research and wrote the manuscript. J.B., B.A.K.K., and C.M.E.K. designed the experiments and analyzed the data. B.A.K.K., C.M.E.K., Y.F. measured NMR; B.A.K.K., C.M.E.K., and Y.F. performed the HPLC experiments. H.S.C. performed experiments and managed the revision process. G.H. and M.M. devised the reaction‐diffusion model and revised the manuscript.

## Conflict of Interests

The authors declare no conflict of interest.

## Supporting information



Supporting Information

## Data Availability

The data that support the findings of this study are available from the corresponding author upon reasonable request.
