# Supplementary material for: Chemically Fueled Active Transport
Source: Angew Chem Int Ed Engl. 2025 May 28;64(27):e202500243. doi: 10.1002/anie.202500243 (PMC12207377; doi:10.1002/anie.202500243)
Supplement: Supplementary file 1 — Supporting Information [file ANIE-64-e202500243-s001.pdf]

Supporting Information for

Chemically fueled active transport.

Christine M. E. Kriebisch,[a] ‡ Brigitte A. K. Kriebisch,[a] ‡ Gregor Häfner,[b][c] Héctor Soria-Carrera,[a] Yanyan Fei,[a] Marcus Müller,[b][c] and Job Boekhoven*[a]

‡ These authors contributed equally.

[a] C. M. E. Kriebisch, B. A. K. Kriebisch, H. Soria Carrera, Yanyan Fei, Prof. Dr. J. Boekhoven,
School of Natural Sciences
Department of Bioscience
Technical University of Munich
Lichtenbergstraße 4, 85748 Garching, Germany
E-mail: job.boekhoven@tum.de

[b] Dr. G. Häfner, Prof. Dr. M. Müller
Institute for Theoretical Physics
Georg-August University
Friedrich-Hund-Platz 1, 37077 Göttingen, Germany

[c] Dr. G. Häfner, Prof. Dr. M. Müller
Max Planck School Matter to Life
Jahnstraße 29, 69120 Heidelberg, Germany

The PDF file includes:

Materials and Methods

Supporting Notes

Figures S1 to S20

Tables S1 to S8

Schemes S1 to S5

References

MATERIALS AND METHODS

Materials

Acetonitrile (ACN) (High-performance liquid chromatography (HPLC grade), 1-pentylamine, Cbz-D, Cbz-D anhydride (BLD pharma), chloroform (HPLC grade), deuterated dimethyl sulfoxide (DMSO-d_6), deuterated water (D_2O), dichloromethane (DCM), 1-ethyl-3-(3-dimethylaminopropy(L)carbodiimide hydrochloride (EDC × HCl), hydrochloric acid, 4-morpholineethanesulfonic acid (MES) buffer, Nile Red, Nitro-phthalic acid, sodium hydroxide (NaOH), Methoxy phthalic acid, Phthalic acid, Phthalic anhydride, Sodium trimethylsilylpropanesulfonate (DSS, TCI chemical), Sulfo-phthalic acid (TCI chemical), trifluoroacetic acid (99%, TFA) were purchased from Sigma-Aldrich unless indicated otherwise. All chemicals were used without any further purification unless indicated otherwise.

Methods

General sample preparation. Stock solutions of the 20 mM Cbz-D, 50 mM sulfo-phthalic acid and 50 mM nitro-phthalic acid were prepared in 200 mM MES buffer at pH 6. The pH of the peptide stocks was adjusted to pH 6. 3 M EDC stock solutions were prepared freshly in 200 mM MES buffer at pH 6. 400 mM 1-pentylamine stocks were prepared freshly in MQ water. 200 mM MES buffered water was prepared by dissolving the MES hydrate in MQ water. The pH of the 200 mM MES buffered water was adjusted to pH 6, using 5 M and 1 M sodium hydroxide solution. 20 mM DSS standards are prepared in D_2O. The stock solutions were stored at 8 °C in the fridge until further use.

Synthesis of EDU. We synthesized EDU by hydrolyzing 1-ethyl-3-(3-dimethylaminopropycarbodiimide hydrochloride (EDC × HCl) at room temperature for one month in MQ water. We lyophilized EDU and used it without further purification.

^{1}H-NMR spectroscopy. ^{1}H-NMR spectra for passive transport experiments were conducted on Bruker AV400US (400 MHz). Chemical shifts are given as δ-values in parts per million (ppm) relative to the internal standard DSS (20 mM) in D_2O (δH: 1.00) or the deuterated solvent peak: DMSO-d_6 (δH: 2.50). For the denotation of the observed signal multiplicities, the following abbreviations were used: s (singlet), d (doublet), dd (doublet of doublets) t (triplet), and m (multiplet).

^{1}H-NMR spectroscopy was used to determine the passive transport of Cbz-D (precursor), EDC (fuel), and EDU (waste). We recorded ^{1}H-NMR with water suppression and calculated the EDC, EDU, concentration by comparing the integral of Cbz-protons at 7.48 – 7.34 ppm, the integral of CH_3 group of EDC at 1.14 - 1.10 ppm, the integral of CH_3 group of the 1-[3-(dimethylamin(O)propyl]-3-ethylurea (EDU, Scheme S1) at 1.08 – 1.03 ppm to the integral of the internal DSS standard at 0.0 ppm. MestReNova© software (Version 11.0.0.-17609) was used to analyze all recorded NMR spectra.

Scheme S1: Monitored CH_3-group in the 1H-NMR measurements of **(A)** EDC, **(B)** EDU.

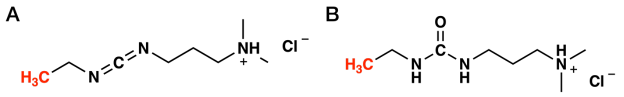

Analytical reversed-phase high-performance liquid chromatography (HPLC). Concentration profiles of the chemical reaction systems were monitored by the analytical reversed-phase HPLC (HPLC, Thermo Fisher Dionex Ultimate 3000, Hypersil Gold 250 × 4.8 mM) applying a linear gradient of ACN (2% to 98%) and water each with 0.1% TFA. We monitored the kinetic profiles as described previously,[1-2] applying the quenching method described by Schnitter and Boekhoven (2020)[1] to irreversibly convert the anhydride into a monoamide, which we call alkylamide.

Kinetic measurements.

Reaction kinetics. We started the reaction cycle by adding EDC to the Cbz-D/nitro-phthalic acid/sulfo-phthalic acid precursor. The total reaction volume was 600 µL. At a pre-determined time, we took 20 µL aliquots and quenched them with freshly prepared 20µL of 400 mM 1-pentylamine to stop the reaction by increasing the pH and thereby irreversibly converting the anhydride into a monoamide. We injected 10 µL of the quenched solution without further dilution (Fig. S1).

Anhydride hydrolysis. We dissolved 10 mM Cbz-D-anhydride and 10 mM phthalic anhydride in chloroform that was saturated with 200 mM MES buffer pH 6. At a pre-determined time, we took 5 µL aliquots and quenched them with freshly prepared 15µL of 400 mM 1-pentylamine in chloroform to stop the reaction by irreversibly converting the anhydride into a monoamide. We evaporated the chloroform and dissolved the residue in 20µL freshly prepared 400 mM 1-pentylamine in MQ water. We injected 7 µL of the quenched solution into the HPLC and measured the anhydride hydrolysis over three days.

Active and passive transport. The U-tube was filled with 500-1000 µL Chloroform, and a stirring bar was added. 100-800 µL 200 mM MES pH 6 was added to the receiver. The final amount of EDC (active transport)/ EDU (passive transport) was added to the reaction solution, and 100-800 µL reaction solution was added to the sender. The U-tube was positioned on the stirring plate (1000 rpm or 1600 rpm). The U-tube was tightly closed with Parafilm. For refuel experiments, we refueled the sender phase with a finite amount of EDC at a pre-determined time. At a pre-determined time, we took 5 µL or 15 µL aliquots and quenched them with freshly prepared 15 µL of 400 mM 1-pentylamine. We injected 10 µL or 7 µL of the quenched solution without further dilution (Fig. S1).

To measure the chloroform composition, we took 5 µL aliquots and quenched them with freshly prepared 15µL of 400 mM 1-pentylamine in chloroform to stop the reaction by irreversibly converting the anhydride into a monoamide. We evaporated the chloroform and dissolved the residue in 20 µL freshly prepared 400 mM 1-pentylamine in MQ water. We injected 7 µL of the quenched solution into the HPLC and measured the chloroform composition.

UV-Vis spectroscopy. We measured the absorbance at 600 nm as a function of time when fueling a solution of 10 mM Cbz-D/ 10 mM sulfo-phthalic acid/ 10 mM nitro-phthalic acid with EDC to confirm that it is not assembling at our conditions. The samples were prepared in 96-UV transparent well plates with a sample volume of 100 µL.

Liquid chromatography – mass spectrometry (LC-MS). LC-MS experiments were conducted on LCQ Fleet Ion Trap Mass Spectrometer (Thermo Scientific). All samples were analyzed in positive mode, and analysis was performed using the Thermo Xcalibur Qual Browser 2.2 SP1.48 software LCQ Fleet Ion Trap Mass Spectrometer. 1 µL of the samples were directly injected into an analytical reversed-phase C18 column upstream of the 3D ion trap (LCQ Fleet Ion Trap Mass Spectrometer) without further dilution. The samples were tracked by mass in positive mode, and with a UV/Vis detector at 220 nm and 280 nm. All compounds involved were separated using linear gradients of water and acetonitrile from 95:5 to 5:95 in 8 min and applying a flow rate of 0.7 mL min^{-1}. Both eluents contained 0.1% formic acid.

SUPPORTING NOTES

Statistical analysis

We fitted curves using the SciPy package.[2] We used the Python package Pingouin for statistical analysis.[3] In the manuscript text, the errors are standard deviation.

Reaction model for systems with homogeneous concentration

We start out by describing the reaction kinetics for a homogeneous system and generalize it to a reaction-diffusion model later. While we analyzed a range of different precursor molecules, the reaction pathways, as well as the resulting kinetics are qualitatively the same for all of them. For all the described reactions it is a good approximation to assume that these are irreversible. In addition, their kinetics are well described by mass-action kinetics, *i.e.*, the forward reaction flux is proportional to the product of reactant concentrations. [7,8] This choice of reaction kinetics is justified in our case because the change in internal molecular energy is large. For instance, EDC is a molecule of high internal energy, whereas EDU is not. This becomes clear in the time evolution since the EDC concentration vanishes in the long-time limit. The same holds true for other molecules in the reaction cycle.

Let us briefly describe how the thermodynamically consistent description of reaction kinetics is simplified to mass-action kinetics, on the example of a binary reaction. For the sake of simplicity let us take the reaction $A + B \rightleftharpoons C$ with two reactants A, B and one product C. In the simplest form, the chemical potential of each molecular species takes the form

$$\mu_I = k_B T \, ln[I] + w_I \qquad (1)$$

for $I = A, B, C$, the Boltzmann constant k_B, temperature T and the enthalpy w_I. Thereby the first term accounts for the translational entropy and the second one can be interpreted as the internal molecular energy. Notice that there are no interactions among species, but the argument is qualitatively the same with interactions, except that the internal molecular energies become concentration dependent. The time evolution of concentrations, $[I]$, follows the differential equation

$$\frac{d[C]}{dt} = -\frac{d[A]}{dt} = -\frac{d[B]}{dt} = \mathrm{s} \qquad (2)$$

where s is the net reaction flux, i.e., the sum of forward and backward reaction fluxes, in accord with detailed balance (see work of Zwicker, Ref. [8])

$$s = \tilde{\mathrm{k}}\left[\exp\left(\beta(\mu_A + \mu_B)\right) - \exp(\beta\mu_C)\right] \qquad (3)$$

$\tilde{k}$ is a kinetic coefficient that determines the rate at which reactions occur. Using the chemical potentials, we obtain

$$\begin{aligned} s &= \tilde{k}\left[[A][B]\exp(\beta(w_A + w_B)) - [C]\exp(\beta w_C)\right] \\ &= k[A][B]\left(1 - \frac{[C]}{[A][B]}\exp\left(\beta(w_C - w_A - w_B)\right)\right) \\ &= k[A][B] - k\frac{[A]^\infty[B]^\infty[C]}{[C]^\infty} \approx k[A][B] \qquad (4) \end{aligned}$$

Here, we used the definition $k = \tilde{k}\exp\left(\beta(w_A + w_B)\right)$ and introduced the equilibrium concentrations $[I]^\infty$. The approximate final equality is true in the special case that the enthalpies of the reactants are far larger than the one of the product, $w_A + w_B \gg w_C$, and the reactant concentrations are finite. To be precise, the latter condition does break down in the long-time limit, where irreversible reaction dynamics lead to vanishing reactant concentrations, while in the thermodynamically consistent variant, they adopt finite, yet vanishingly small values. Before going into the details of the specific reactions at hand, the irreversible dynamics are good approximation in our case, because the concentrations of fuel (EDC) and product (anhydride) approximately vanish in the long-time limit, see for instance Figure S3. In the thermodynamically consistent formulation, the equilibrium condition for the reaction flux to vanish $s_I = 0 \quad \Leftrightarrow \frac{[A]^\infty[B]^\infty}{[C]^\infty}\exp(\beta(w_C - w_A - w_B))$ dictates finite concentrations of reactants in the long-time limit. However, for the results of this work, there is no difference between a vanishing and a negligibly small fuel concentration. This justifies the use of irreversibility and mass-action kinetics in our case.

The time evolution of concentrations of all relevant components in the reaction cycle is based on four ordinary differential equations (ODEs), which describe five chemical reactions (*vide infra*): 1) direct hydration of fuel (EDC) (k_0), 2) the activation of acid to the intermediate O-acylisourea (k_1), 3) the spontaneous hydrolysis of O-acylisourea (k_2), 4) the formation of anhydride *via* the intramolecular reaction of intermediate O-acylisourea with the second carboxylate of the acid (k_3), and 5) the spontaneous hydrolysis of anhydride (k_4).

Reaction 0 (k0):

EDC hydrates directly with a first-order rate constant of 0.00042 min^{-1} at pH 6, as determined by HPLC.

Scheme S2: Reaction 0 (k_0).

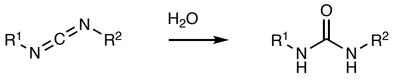

Reaction 1 (k_1):

Fuel activates acid to its corresponding O-acylisourea in a second-order reaction.

Scheme S3: Reaction 1 (k_1).

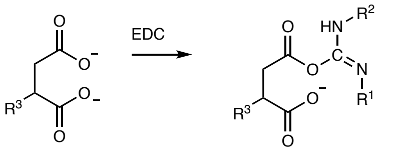

Reaction 2 (k_2):

The O-acylisourea spontaneously hydrolyzes in a pseudo-first-order reaction.

Scheme S4: Reaction 2 (k_2).

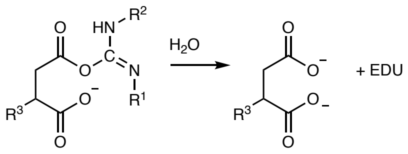

Reaction 3 (k_3):

O-acylisourea reacts with the second carboxylate of the acid to form the intramolecular anhydride.

Scheme S5: Reaction 3 (k_3).

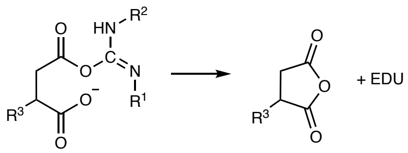

Reaction 4 ($k_{hyd} = k_4$):

The anhydride spontaneously hydrolyzes in a pseudo-first-order reaction.

Scheme S6: Reaction 4 ($k_{hyd} = k_4$).

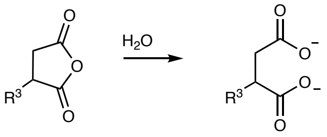

Set of ordinary differential equations (ODEs)

The combination of the above reactions leads to the following set of ODEs to describe the system. Every reaction gives rise to a term proportional to the product of the reactants' concentrations and the proportionality factor is the reaction rate k, as defined above. Ac, refers to acid, An refers to anhydride, F refers to fuel, W to waste, and In to the intermediate product of O-acylisourea. The full set of equations, that accounts for each of the above reactions reads

$$\frac{d[F]}{dt} = -k_0[F] - k_1[F][Ac] \tag{5}$$

$$\frac{d[Ac]}{dt} = -k_1[F][Ac] + \mathrm{k}_2[In] + k_4[An] \tag{6}$$

$$\frac{d[In]}{dt} = k_1[F][Ac] - (k_2 + \mathrm{k}_3)[In] \tag{7}$$

$$\frac{d[An]}{dt} = \mathrm{k}_3[In] - k_4[An] \tag{8}$$

$$\frac{d[W]}{dt} = k_0[F] + k_1[Ac][F] \tag{9}$$

Now we assume that reactions, involving the intermediate product occur on small time scales, such that it's concentration quickly adopt its stationary value

$$[In] = \frac{k_1[F][An]}{k_2+k_3} \tag{10}$$

This reduces the set of differential equations to two, describing acid, anhydride, fuel and waste, where the evolution of the first two and the last two are redundant

$$\frac{d[F]}{dt} = -\frac{d[W]}{dt} = -k_0 * [F] - k_1 * [F] * [Ac] \tag{11}$$

$$\frac{d[Ac]}{dt} = -\frac{d[An]}{dt} = -k_1 * [F] * [Ac] + \frac{k_1 * k_a * [Ac] * [F]}{k_a+1} + k_4 * [An] = -k_{\text{fuel}} * [F] * [Ac] + k_{\text{hyd}} * [An] \tag{12}$$

In the last equations, we used the definitions, $k_a = \frac{k_2}{k_3}$, $k_{\text{fuel}} = \frac{k_1}{k_a+1}$ and $k_{\text{hyd}} = k_4$, simplifying the equations.

Least-square fitting of the kinetic parameters and simulation of concentration profiles was devised in Python (Table S2, 3), as described by Chen *et al.*[4] and Hartley's work.[5] We updated the Python script described in Chen *et al.*,[4] for the differential equations described above to fit the experimental data. This results in the kinetic model parameters given in Table S2.

Since $k_a = k_2/k_3 \leq 10^{-6} \ll 1$ is vanishingly low, and therefore $k_{\text{fuel}} \approx k_1$,the system is in line with an intramolecular process.[4, 6] Hence, fitting the same system with the constraint $k_{\text{fuel}} = k_1$, results approximately in the same parameters (Table S3). In the following reaction-diffusion model, we are using the results without this constraint.

Python codes

We used the previously described Python script from Chen et al.[4] to fit the experimental data and update it for our set of ODEs. The code solves the ODE system and fits it to the experimental data, minimizing an error function using the lmfit package (https://zenodo.org/record/11813). We further minimized the error of the kinetic model parameters by performing a bootstrap with five iterations in the fitting that generated a distribution of kinetic constants. For the fitting, we used the median of the distribution to be more robust towards outliers compared to the mean. We used the root squared mean error of the fitting to calculate the 95 % confidence interval. We evaluated the goodness of fitting by calculating the R^2 error.

We obtained the kinetic model parameters by fitting the kinetic profiles of fuel, acid, and anhydride for one set of typical reaction conditions. We validated the obtained kinetic constants by predicting the data set at different fuel levels. The set of rate constants fitted those reasonably well (Table S2, 3).

Reaction-diffusion model

In order to capture the diffusive dynamics, when the aqueous phase neighbors a chloroform phase and transport across the interface may occur, we make use of a one-dimensional continuum model to capture the reaction-diffusion dynamics of the different molecular species, as well as their partitioning in the aqueous and chloroform phase, respectively. To this end, we consider the local concentration of the EDC fuel (F), the precursor acid (Ac), and the product anhydride (An), denoted by $[I](x,t)$ for $I = Ac, An, F$ in one spatial dimension, x, and time t. We consider molecular diffusion in the sender and receiver phases. In the chloroform phase, which is externally stirred in the experiment, the concentrations are homogeneous, such that the transport across this phase is dictated by the equilibration rate at the interfaces. This principle has been tested experimentally, for instance, by varying the chloroform phase length, which would result in significant differences in efficiency if the transport across the chloroform phase was driven by diffusion.

All in all, we model the three phases – sender, chloroform, and receiver, separated by sharp interfaces that molecules cross at a finite rate. Within the sender and receiver phases, the time evolution of the concentration fields follows a modified diffusion equation that includes the same reaction kinetics as the reaction model described above, as well as interface equilibration at a finite rate

$$\frac{\partial [I]^X(x,t)}{\partial t} = D\,\frac{\partial^2 [I]^X(x,t)}{\partial x^2} - s_I^X(x,t) + r_I^X \delta(x - x_{XC}) \qquad (9)$$

where the superscript, $X = S, R$, denotes the fields and values in the respective phases, D denotes the diffusion coefficient and x_{XC} is the position of the interface between the phases X and X'. The chemical reactions are introduced as sources and sinks s_c, following the model for simulating and fitting the set of ODEs introduced for the homogeneous test systems. Specifically, in the sender and receiver phases $X = S, R$, we take

$$\begin{aligned} s_F^X &= -k_0[F]^X - k_1[F]^X[Ac]^X \\ s_{Ac}^X &= +k_{\mathrm{hyd}}[An]^X - k_{\mathrm{fuel}}[F]^X[Ac]^X \\ s_{An}^X &= -k_{\mathrm{hyd}}[An]^X + k_{\mathrm{fuel}}[F]^X[Ac]^X \end{aligned} \qquad (10)$$

All reaction contributions vanish in the chloroform phase since practically no fuel and no water are present $s_I^C = 0$.

In the last term in eq. (5), the factor r_I^X corresponds to the interface relaxation rate, derived as follows. We consider local binary interactions of the molecules with water and chloroform, respectively. Since the molecular concentrations are far smaller than the ones of water and chloroform, the latter concentrations are essentially constant, and therefore, the interactions only add a constant (different in each phase) to the chemical potentials

$$\mu_I^X(x,t) = k_B T \ln[I]^X(x,t) + w_I^X \qquad (11)$$

for $X = S, C, R$, where w_I^X is the local enthalpy of species I in the respective medium, X. Chemical equilibrium now demands that the chemical potentials at the interfaces match

$$\mu_I^X(x_{XC}) = \mu_I^C(x_{XC}) \qquad (12)$$

for $X = S, R$. The difference in interactions of molecules with water and chloroform hence dictates a discontinuity of the concentrations. Fulfilling this equation with the constraint that the total concentrations are conserved,

$$([I]^X)_{XC}^{eq} + ([I]^C)_{XC}^{eq} = [I]^X(x_{XC}) + [I]^C(x_{XC}) \qquad (13)$$

leads to the equilibrium concentrations

$$\begin{aligned} ([I]^X)_{XC}^{\mathrm{eq}} &= \frac{[I]^X(x_{XC}) + [I]^C(x_{XC})}{e^{\beta\Delta\mu_I} + 1} \\ ([I]^C)_{XC}^{\mathrm{eq}} &= \frac{[I]^X(x_{XC}) + [I]^C(x_{XC})}{e^{-\beta\Delta\mu_I} + 1} \end{aligned} \qquad (14)$$

with $\beta = (k_B T)^{-1}$ and the interaction difference (aka difference in the excess chemical potential) defined by

$$\Delta\mu_I := w_I^X - w_I^{\mathrm{C}}. \qquad (15)$$

This is taken the same for the sender and receiver phase $w_I^{\mathrm{R}} = w_I^{\mathrm{S}}$. If $\Delta\mu_I$ is positive, there is a preference for the chloroform phase, while species i prefers the sender and receiver phases for negative values. Correspondingly, a negative value hinders diffusion of the species across the chloroform phase. We disregard the diffusion of fuel across the chloroform phase, corresponding to the limit scenario $\Delta\mu_{\mathrm{F}} \to -\infty$.

This determines the interface relaxation rate, which is proportional to the difference between the current concentration and the equilibrium concentration

$$\begin{aligned} r_I^X &= -r\left([I]^X - ([I]^X)_{XC}^{\mathrm{eq}}\right) \\ &= -r\,\frac{[I]^X - e^{-\beta\Delta\mu_I}[I]^C}{e^{-\beta\Delta\mu_I} + 1} \end{aligned} \qquad (16)$$

where r is a kinetic coefficient, which we assume to be the same for precursor and product.

In the experiment, continuous stirring leads to a fast transport of the molecules across the chloroform phase. Therefore, we consider this phase to be homogeneous. In the absence of reactions, the concentration of precursor and Cbz-anhydride in the chloroform phase evolves in time according to

$$\frac{\mathrm{d}[I]^C}{\mathrm{d}t} = -\frac{r}{L_C}\,\frac{2e^{-\Delta\mu_I}[I]^C - [I]^S(x_{SC}) - [I]^R(x_{RC})}{e^{-\beta\Delta\mu_I} + 1} \qquad (17)$$

As in the experiments, we start each simulation with homogeneous concentrations in each phase. In the sender phase, EDC and the respective acid concentrations are initialized with finite values, while the Anhydride concentration vanishes initially. All concentrations in the remaining phases are initialized zero, if not specified otherwise. At the sides of the domains, we take reflecting boundary conditions to ensure mass conservation. The equations are integrated numerically and propagated with the FTCS (forward time-centered space) algorithm. Each phase has a length L_X which are dictated by the experimental conditions. The problem is discretized with a spatial step size $\Delta x = 0.01$ cm and temporal step size of $\Delta t = 0.01$ min.

The numerical problem is left with four parameters that are not quantified by experiments, namely diffusion coefficient, D, the two chemical potential differences, $\Delta\mu_{Ac}$ and $\Delta\mu_{An}$, and the kinetic coefficient of interface relaxation, r. Note that the kinetic model parameters of the reactions are already quantified by fitting the set of ODEs described earlier.

Parameter fit

The parameters are determined by a least-square fit to a subset of the experimentally measured time evolutions of anhydride concentrations in the receiver phase and standard deviations of the fitted parameters are obtained by boot-strapping. To fit the coefficients for the systems with Cbz-D, we take the time evolution for $L_S = 3.05$ cm with no input of EDC, c^S_{EDC} (t = 0) = 0, to properly evaluate the interaction difference of the precursor and its resulting diffusion through the chloroform phase. In addition, we take the time evolution for finite starting concentration of EDC, $c^S_{\mathrm{EDC}} = 100$ mM, for various sender-phase lengths, $L_S = 0.35$ cm, 1.06 cm, 2.54 cm, 3.05 cm. For all other species of precursor molecules, we take a single sender-phase length of L_S = 0.35cm in addition to the data without input of EDC. These results are compiled in Table S3.

Time evolution in the reaction-diffusion model

We start with example values $L_S = L_R = 3.05$ cm and $L_C = 2.54$ cm, as well as the fit parameters for Cbz-D as the precursor. For these parameters, an increase of the precursor concentration in the receiver phase is observable over several days (Fig. 2C). However, the reactions occur on the order of tens of minutes; the initially provided fuel is consumed within the first two hours, and the product decays in the sender phase on a similar time scale. The product diffuses into the chloroform phase in this time frame and gradually leaks out of the chloroform phase over the coming days.

The local concentrations in the sender phase during the first two hours, as well as the mean concentrations in the sender and chloroform phase are depicted in Fig. S10. While the mean fuel concentration is rapidly decreasing, the anhydride product concentration surges in the first 10 minutes. While it is high and the product concentration in the chloroform phase is low, product enters the chloroform phase, depleting its concentration in the sender phase near the interface. Only molecules that are activated close to the interface can diffuse into the chloroform phase within their life time, t. Hence, the depletion of concentration is visible in the concentration profiles within a distance of $\sqrt{Dt}$ = 0.2 cm. Activation reactions that occur farther away from the interface can be identified as inefficient, *i.e.*, such molecules only diffuse into the chloroform phase with a low probability.

While mean concentrations of anhydride and EDC in the sender phase decay to zero in the long-time limit, the precursor acid concentration does not recover to its initial value because a significant part of the product and a marginal part of the precursor have entered the chloroform phase. This can be observed in the mean concentrations in the chloroform phase. Fuel does not enter the chloroform phase in our reaction-diffusion model. The precursor concentration reaches a stationary value of $[Ac]^C \approx 4 \cdot 10^{-5}$ mM in the chloroform phase. Finally, the mean anhydride concentration rises rapidly in the first hour and afterward gradually decreases over a few days. Once most of the anhydride in the sender phase has hydrolyzed, the anhydride is gradually released from the chloroform phase, almost symmetrically into both the sender and receiver phases. The slow release of the anhydride into the receiver, in combination with fast hydrolysis in the receiver phase into the precursor acid state, *Ac*, results in a slow increase in precursor concentration in the receiver phase, $[Ac]^R$.

Efficiency and turnover time

As in the experiments, we define efficiency as the number of precursor molecules that have (additionally) reached the receiver phase per fuel molecule given into the sender phase. This yields the definition

$$\eta(t) = \frac{\int_R \mathrm{d}x \left([Ac]^R(x,t) - [Ac]\text{^}R(x,t=0)\right)}{\int_S \mathrm{d}x\, [\mathrm{F}]^{\mathrm{S}}(x,t=0)}. \qquad (14)$$

In addition, we obtain the turnover time, as shown in Fig. 4D, from the mean concentration of Cbz-D in the receiver phase. To this end, we fit the simple form $[Ac]^R = [Ac]^R(t \to \infty)\left(1 - \exp(t/t_{to})\right)$, to obtain the turnover time t_{to}. Note that this is only well-defined for a system without passive diffusion, *i.e.* $\Delta\mu_{\mathrm{Ac}} \to -\infty$. In this case, transport is only in the presence of fuel, and the system does not approach the same precursor concentration in the sender and receiver phases within the long-time limit.

The efficiency, $\eta(t)$ is plotted as a function of the anhydride's interaction difference for various time points in Fig. S17 for a system with lengths $L_S = L_R$ =0.35 cm and all other parameters as measured and fitted for Cbz-D as a precursor. The figure includes the two scenarios, without and with passive transport of Cbz-D through the chloroform phase. Without passive transport, one observes a peak in efficiency at vanishing interaction difference. However, from the turnover time in Fig. 4D in the main text, we can observe that the turnover simply increases for large positive interaction differences. This results in a rise in efficiency for times on the order of the turnover time. In the long-time limit, the efficiency approaches a sigmoidal curve that is approximately constant for positive interaction differences. In the scenario with passive transport, the precursor slowly diffuses through the chloroform phase. This increases the efficiency, which rises with time, independent of the interaction difference of the anhydride $\Delta\mu_{\mathrm{Cbz-An}}$. In the long-time limit the curve will approach a constant $\eta \equiv \frac{1}{2}$. However, on the experimentally probed time scales, the effect of passive diffusion

remains small. Note that the effect of passive diffusion is the opposite if the precursor concentration is the same in the sender and receiver phases in the beginning.

SUPPORTING FIGURES

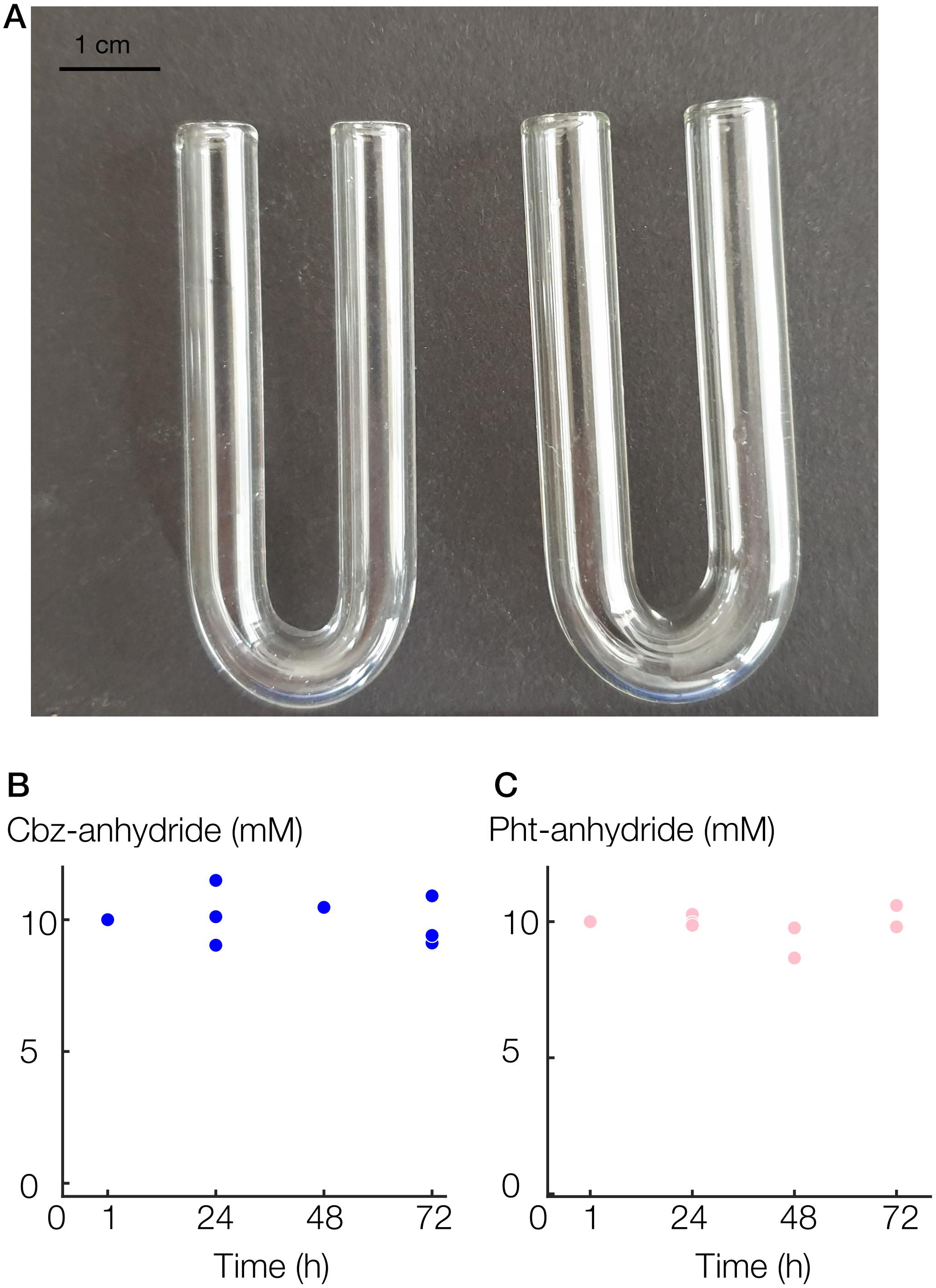

Figure S1. U-tube setup and anhydride hydrolysis in chloroform. (**A**) U-tubes used for the active transport experiments. (**B-C**) Anhydride hydrolysis of (**B**) 10 mM Cbz-anhydride and (**C**) 10 mM phthalic anhydride in wet chloroform that was saturated with the aqueous buffer.

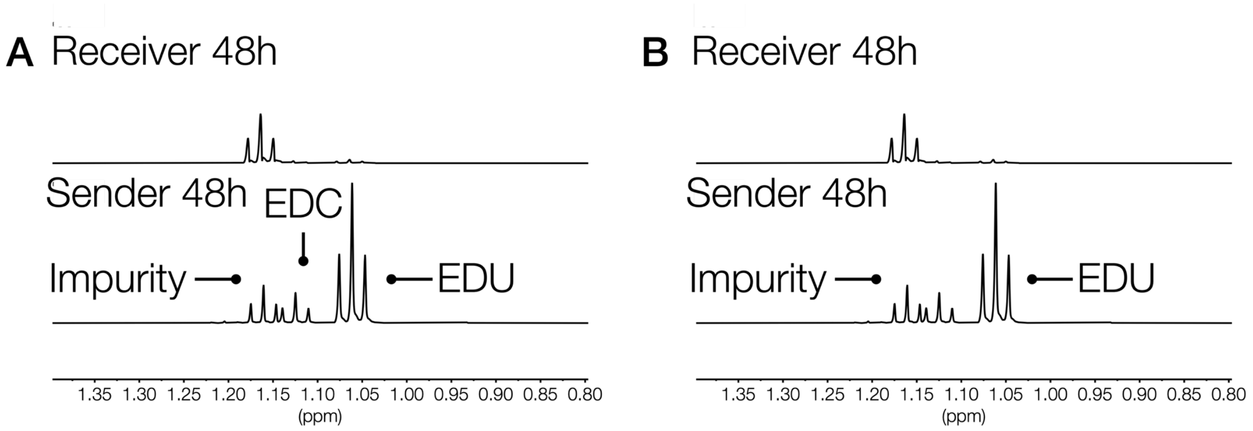

Figure S2. The ^{1}H-NMR spectra of the sender and receiver when fueled with 100 mM EDC after 48 hours show that a bit of EDC and EDU is present on the receiver and passively transports through the chloroform phase.

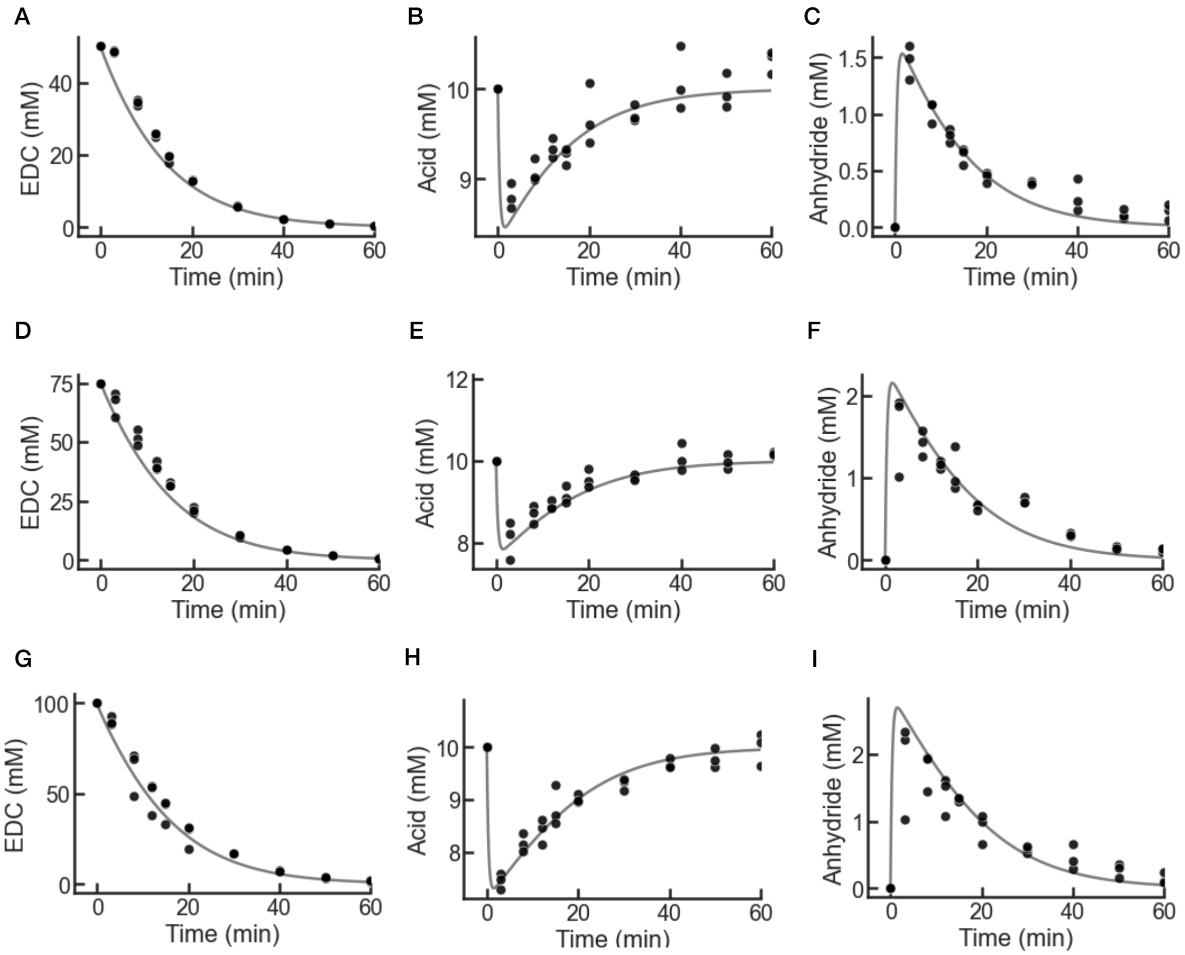

Figure S3. Concentration profiles of 10 mM Cbz-D fueled with 50 mM (A-C), 75 mM (D-F), 100 mM EDC (G-I). (**A, D, G**) EDC profiles. (**B, E, H**) Cbz-D profiles. (**C, F, I**) Cbz-D anhydride profiles. Least-square fitting of the set of ODEs that includes k_a. Lines are fits. Experiments are done in triplicate.

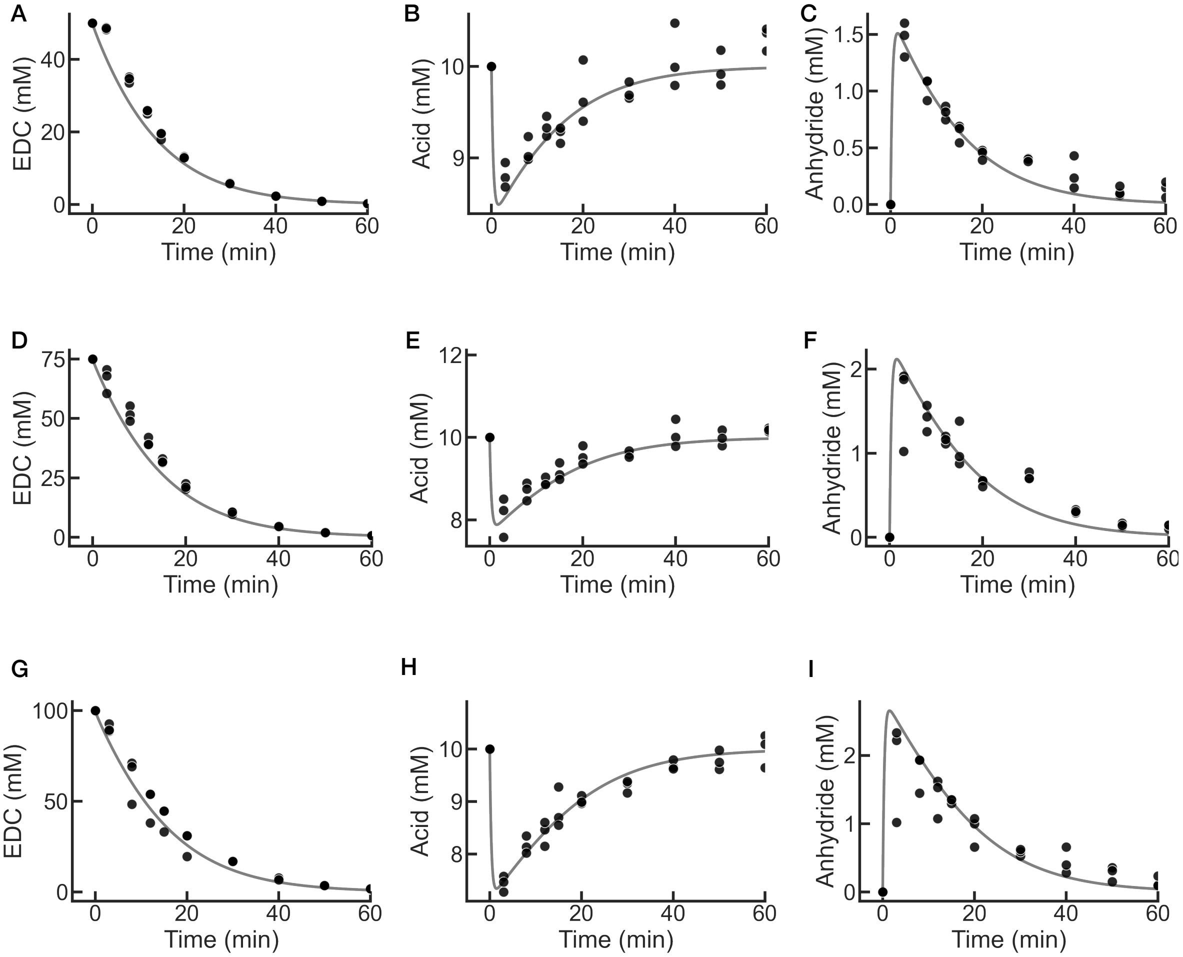

Figure S4. Concentration profiles of 10 mM Cbz-D fueled with 50 mM (A-C), 75 mM (D-F), 100 mM EDC (G-I). (**A, D, G**) EDC profiles. (**B, E, H**) Cbz-D profiles. (**C, F, I**) Cbz-D anhydride profiles. Least-square fitting of the set of ODEs that excludes k_a. Lines are fits. Experiments are done in triplicate.

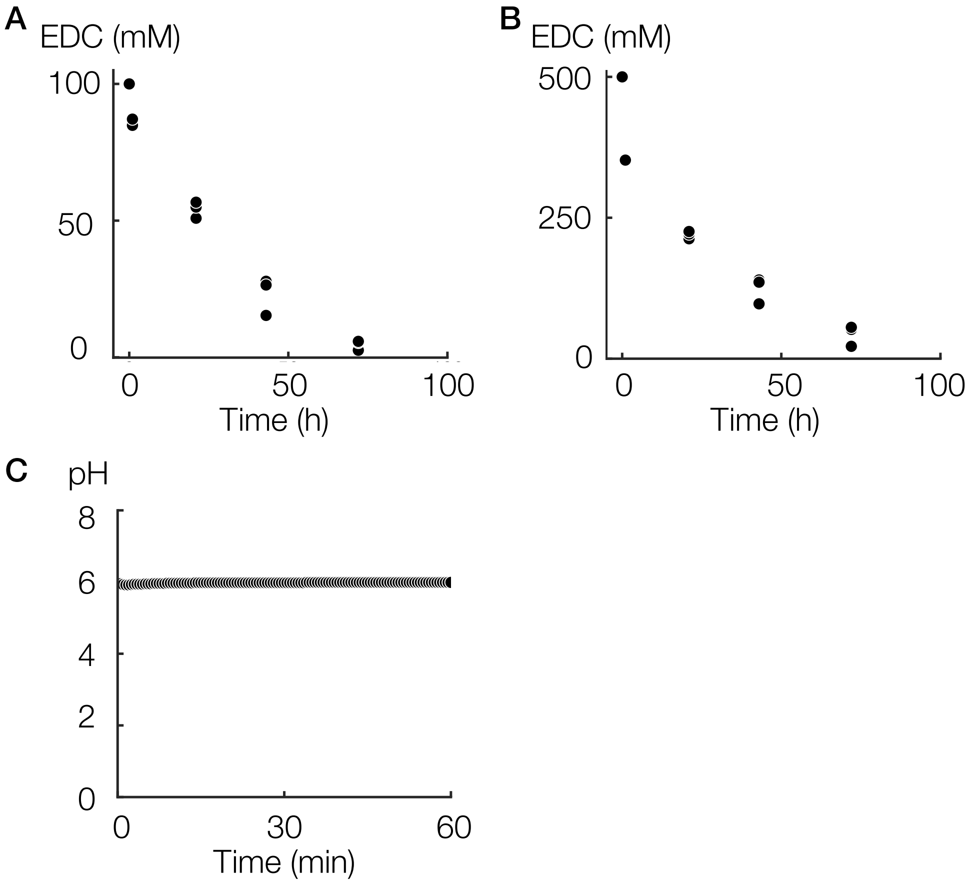

Figure S5. (**A**) 100 mM EDC and (**B**) 500 mM EDC hydration at pH 6, monitored by HPLC (triplicate) and (**C**) pH over time.

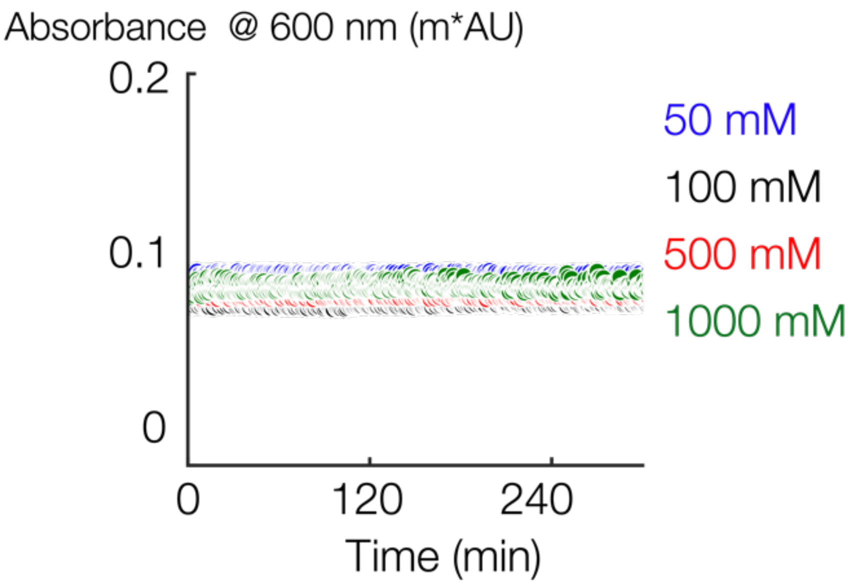

Figure S6. UV-Vis spectroscopy of Cbz-D fueled with 50 mM, 100 mM, 500 mM, and 1000 mM EDC at 600 nm. Experiments were performed in triplicate.

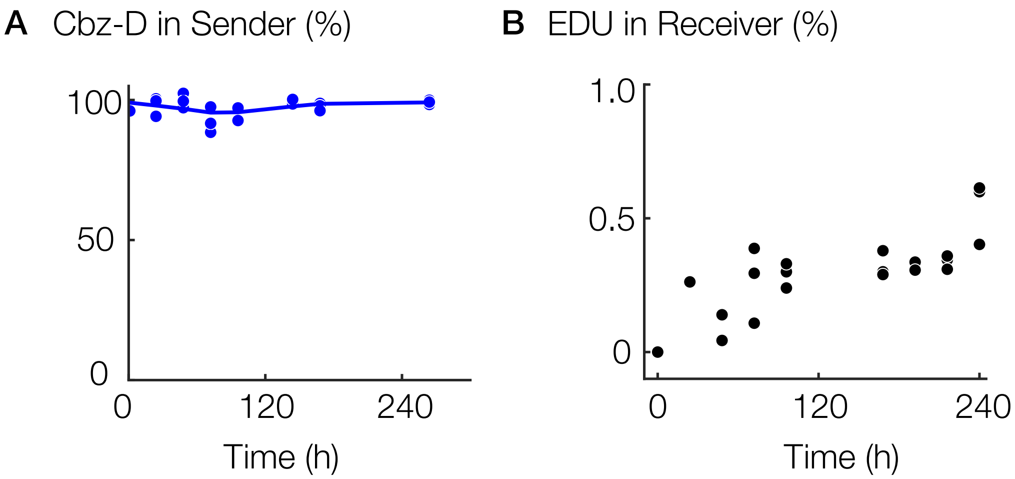

Figure S7. (**A**) Percentage of Cbz-D in sender phase when fueled with 100 mM EDC. The percentage refers to the starting concentration of acid. The line is a guide for the eyes. Experiments were performed in triplicate (sender length = 2.54 cm). (**B**) Percentage of EDU in receiver phase when 10 mM Cbz-D are fueled with 100 mM EDC (sender length = 2.54 cm).

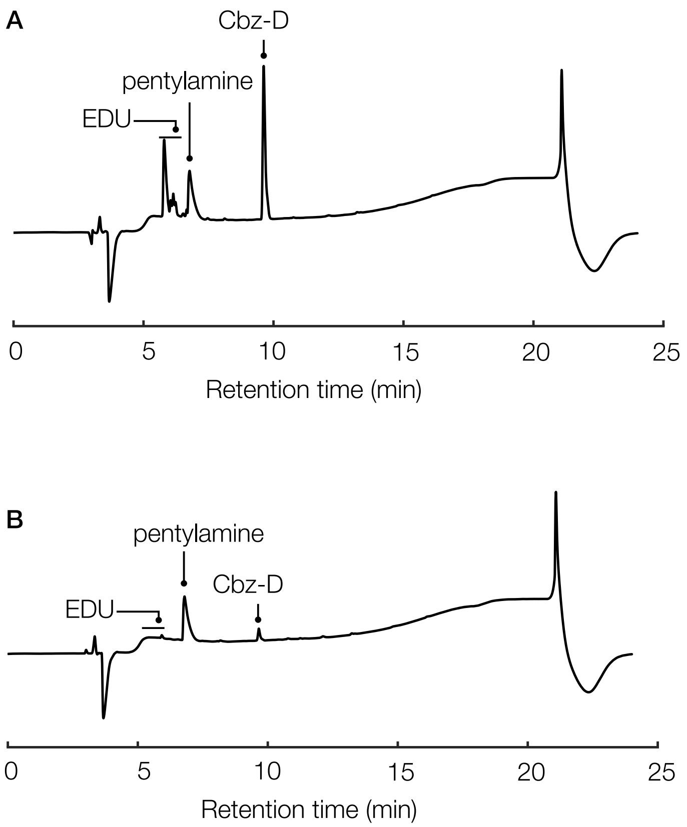

Figure S8. Example of HPLC chromatogram of sender and receiver after 8 days. Example of HPLC chromatogram (absorbance 220 nm) of (**A**) sender and (**B**) receiver when 10 mM Cbz-D are fueled with 100 mM EDC on the sender side and quenched at 8 days with pentylamine.

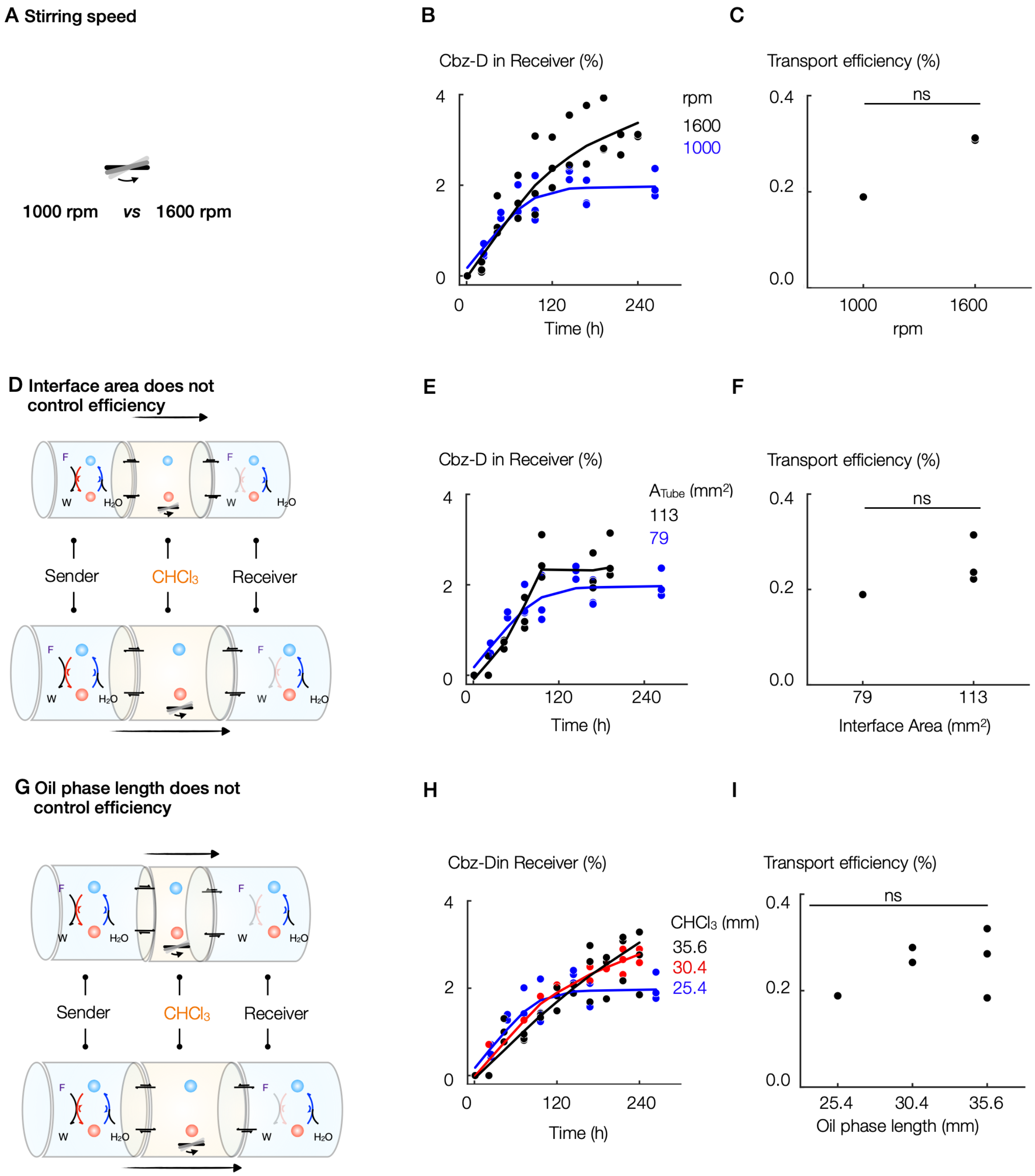

Figure S9. Influence of stirring, interface area, chloroform phase length and sender phase length on the active transport. (**A-C**) Influence of the stirring speed on the transport yield (**B**) and transport efficiency (**C**) of Cbz-D. (**D-F**) Influence of the interface area on the transport yield (**E**) and transport efficiency (**F**) of Cbz-D. (**G-I**) Influence of the chloroform phase length on the transport yield (**H**) and transport efficiency (**I**) of Cbz-D. (**B, E, H**) Lines are guides for the eyes. The percentage refers to the starting concentration of acid. (**C, F, I**) One-way Welch-ANOVA (A-D) *P < 0.05, **P < 0.01, ***P < 0.001, ****P < 0.0001, ns = not significant (P > 0.05). Based on the amount of Cbz-D transported, we calculated the transport efficiency using ($[Cbz\text{-}D]_{receiver}/[Fuel]_{sender}$)×100 %. (**A-F**) Experiments were performed in triplicate using 100 mM EDC, if not otherwise indicated.

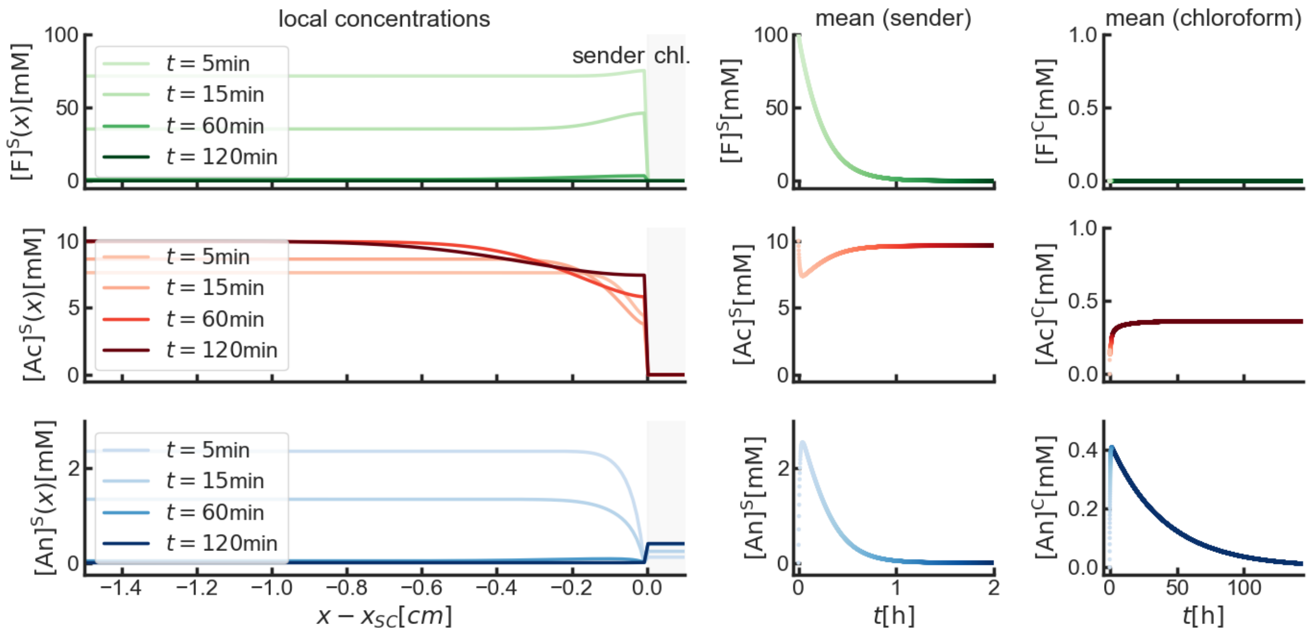

Figure S10. Time evolution of concentrations in the reaction-diffusion model. Left: local concentrations, $[I]^X(x)$, in the immediate vicinity of the sender chloroform phase for different times in the first 2h for EDC (top), Cbz-D (middle) and Cbz-anhydride (bottom). Center: Spatially averaged concentrations in the sender phase in the first 2h. Right: Spatially averaged concentration in the chloroform phase over the first 120h.

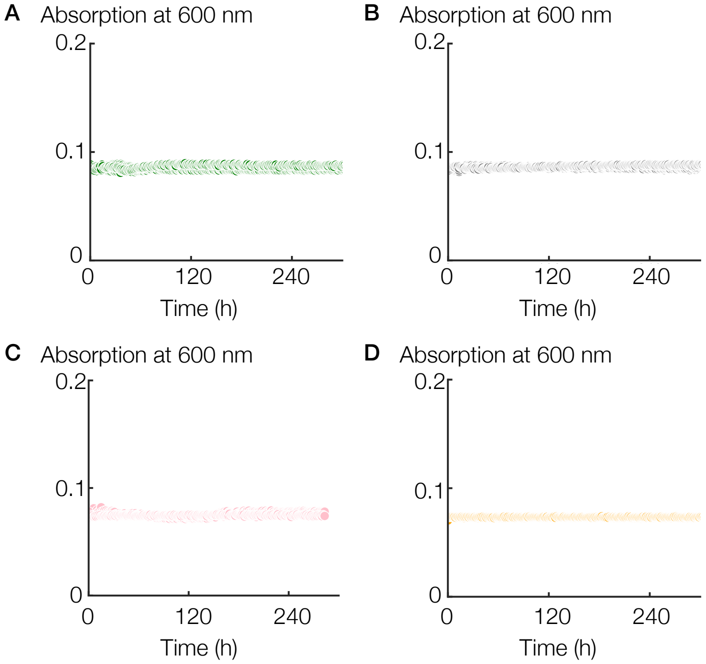

Figure S11. UV-Vis spectroscopy of **(A)** 10 mM sulfo-phthalic acid **(B)** 10 mM nitro-phthalic acid **(C)** 10 mM phthalic acid fueled with 100 mM EDC at 600 nm, and (**D**) 10 mM methoxy-phthalic acid fueled with 5 mM EDC. Experiments were performed in triplicate.

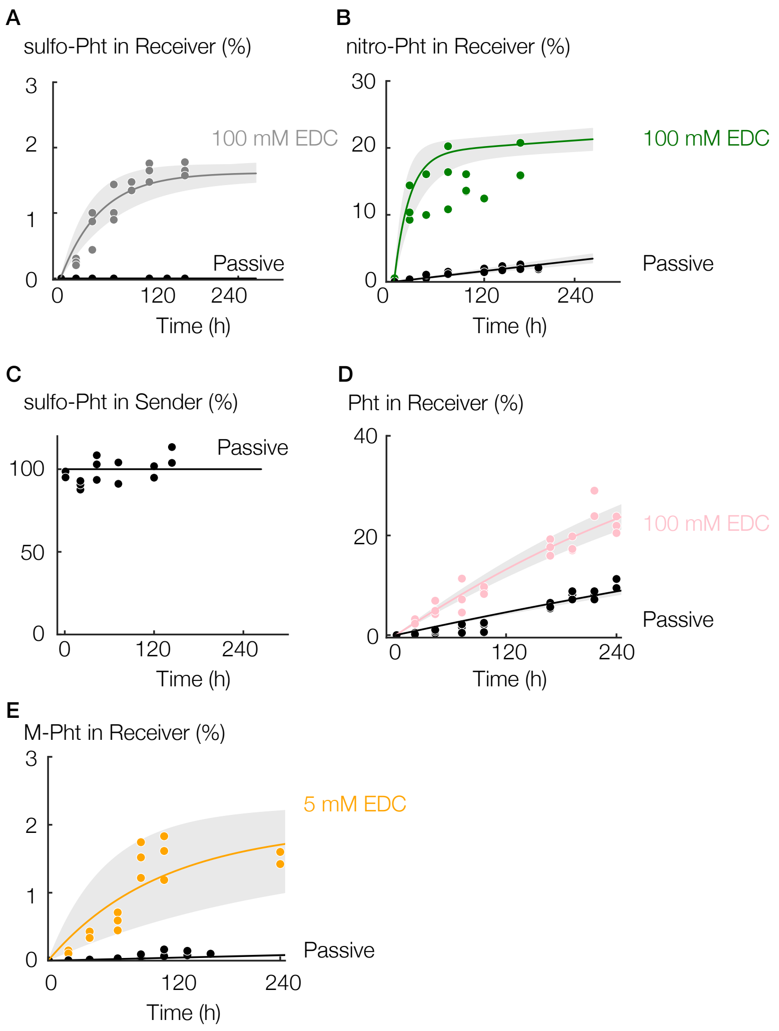

Figure S12. Transport profiles of active and passive transports of sulfo-phthalic acid or nitro-phthalic acid (A, C) Sulfo-phthalic acid profiles. **(B)** Nitro-phthalic acid profiles. In the sender, either 100 mM EDC or 100 mM EDU were added to monitor active or passive transport, respectively. **(D)** Phthalic acid profiles. In the sender, either 100 mM EDC or 100 mM EDU were added to monitor active or passive transport, respectively. **(E)** Methoxy-phthalic acid profiles. In the sender, either 5 mM EDC or 5 mM EDU were added to monitor active or passive transport, respectively. (**A-E**) Curves are predictions by our reaction-diffusion model, where the grey shaded area gives the confidence interval of the predictions. Experiments were performed in triplicate. The percentage refers to the starting concentration of acid.

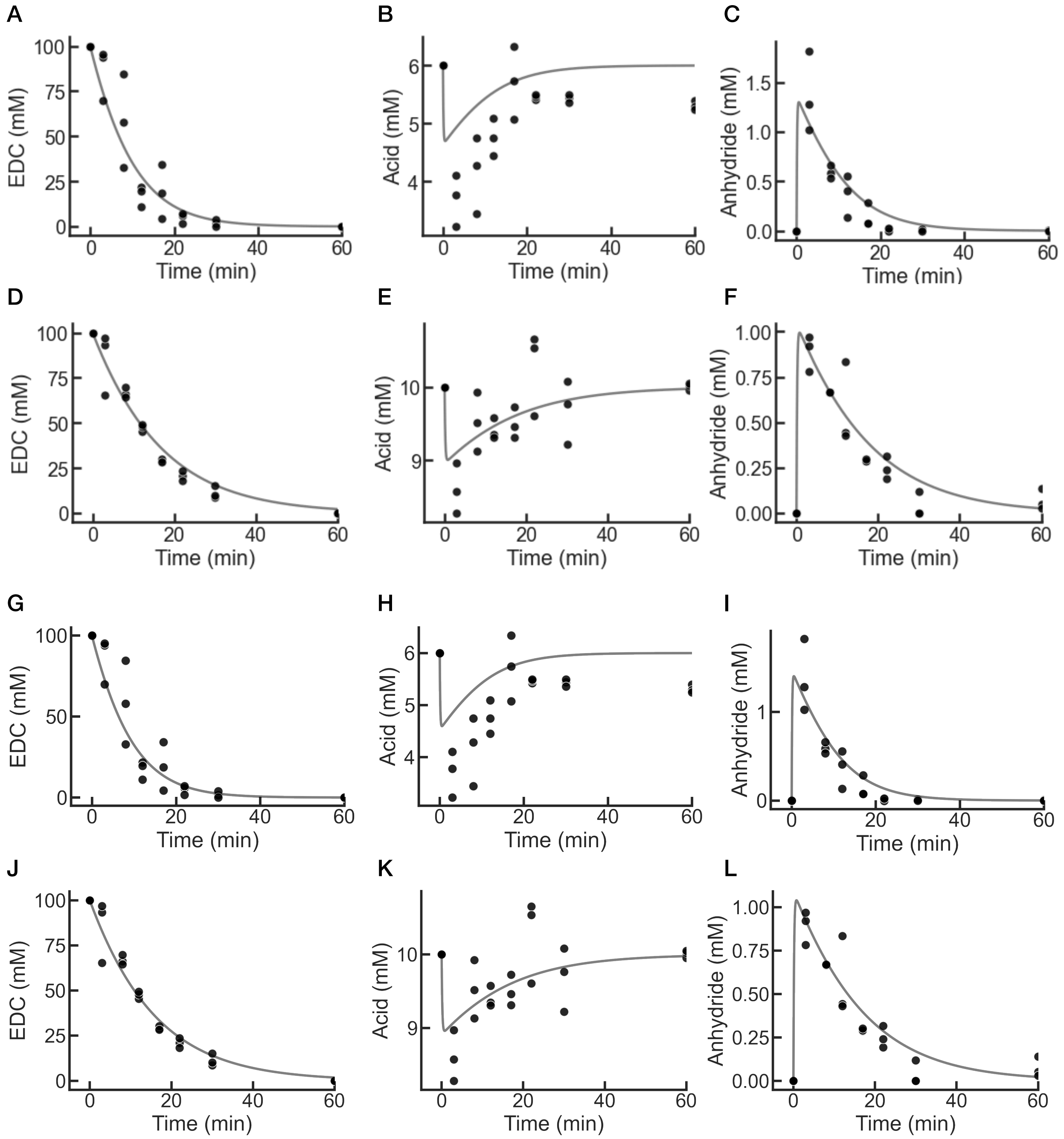

Figure S13. Concentration profiles of 10 mM sulfo-phthalic acid or nitro-phthalic acid fueled with 100 mM EDC. (A, D, G, J) EDC profiles. **(B, H)** Sulfo-phthalic acid profile, **(C, I)** Sulfo-phthalic acid anhydride profile, **(E, K)** nitro-phthalic acid profile, **(F, L)** nitro-phthalic anhydride profile. **(A-F)** Least-square fitting of the set of ODEs that includes k_a, and **(G-L)** excludes k_a. Lines are fits. Experiments are done in triplicate.

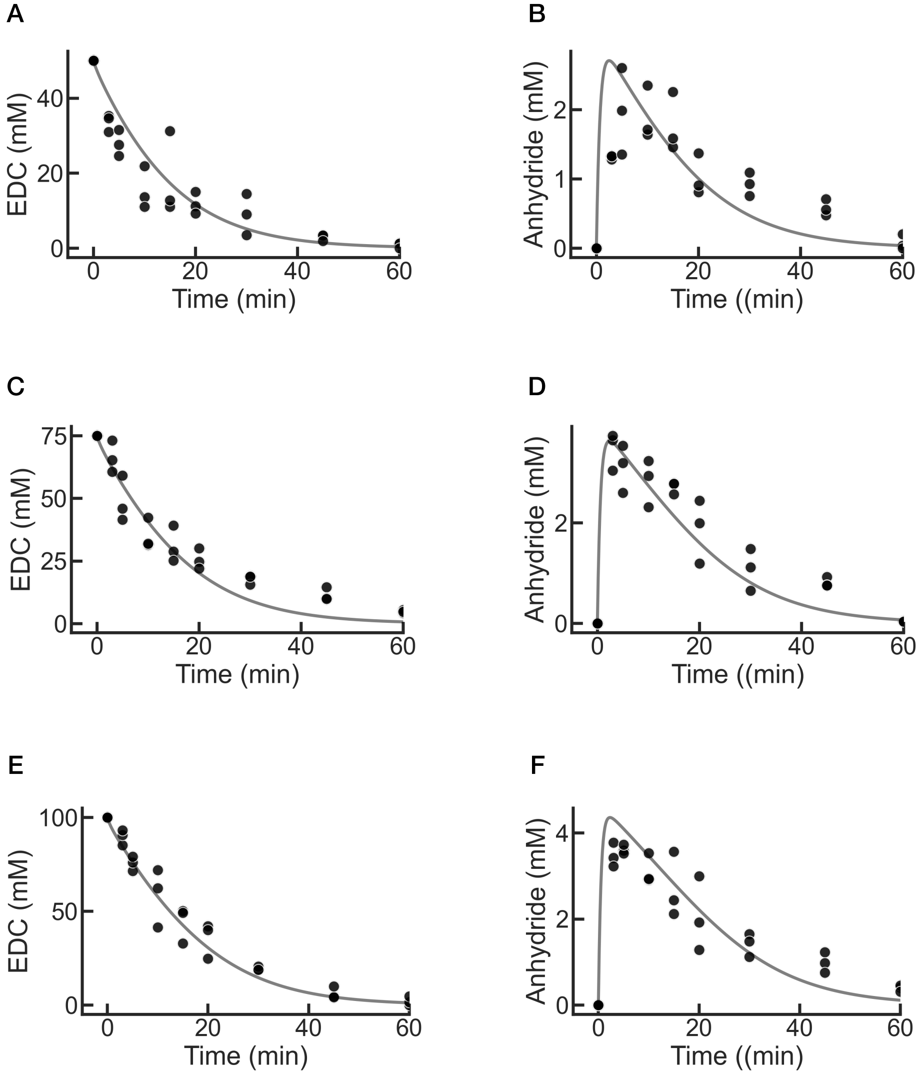

Figure S14. Concentration profiles of 10 mM phthalic acid-fueled with 50 mM (A-B), 75 mM (C-D), or 100 mM EDC (A-F). (A, C, E) EDC profiles and **(B, D, F)** Phthalic anhydride profile. Least-square fitting of the set of ODEs that includes k_a. Lines are fits. Experiments are done in triplicate.

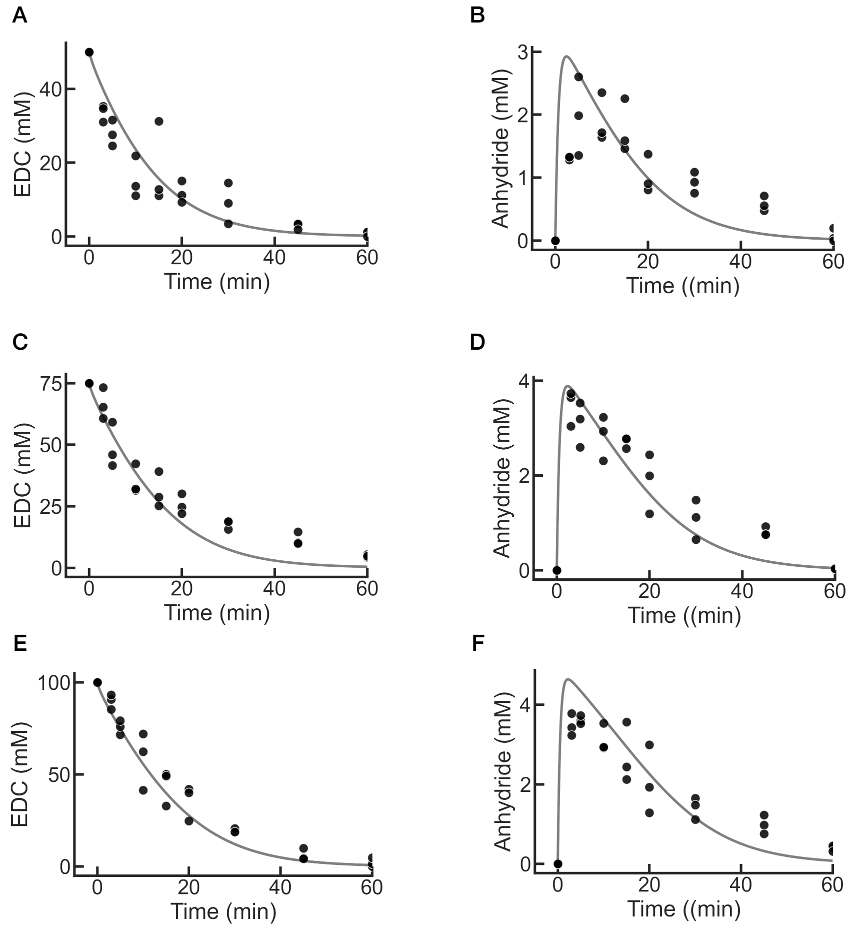

Figure S15. Concentration profiles of 10 mM phthalic acid-fueled with 50 mM (A-B), 75 mM (C-D), or 100 mM EDC (A-F). (A, C, E) EDC profiles and **(B, D, F)** Phthalic anhydride profile. Least-square fitting of the set of ODEs that excludes k_a. Lines are fits. Experiments are done in triplicate.

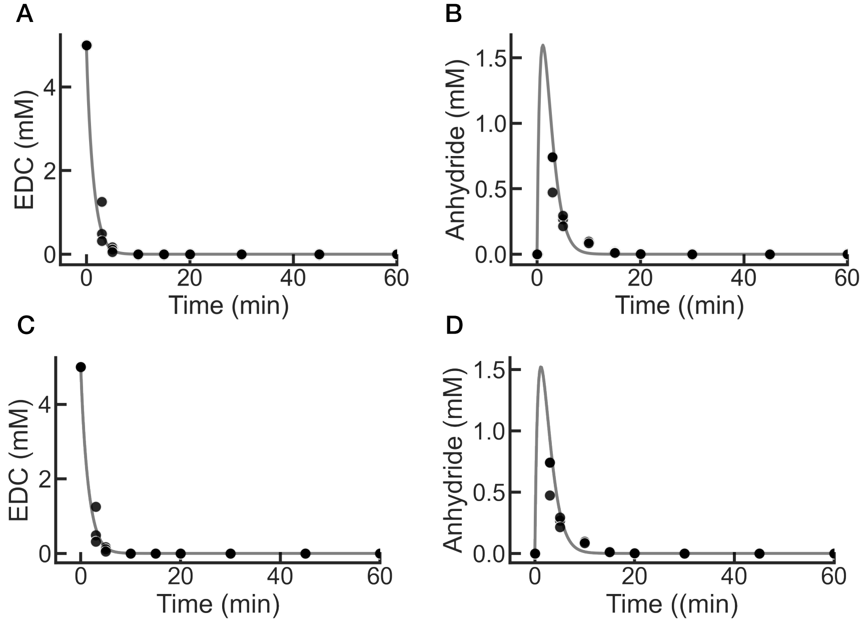

Figure S16. Concentration profiles of 10 mM methoxy phthalic acid-fueled with 5 mM EDC. (A) EDC profile and **(B)** Methoxy-phthalic anhydride profile. **(A-B)** Least-square fitting of the set of ODEs that includes k_a, and **(C-D)** excludes k_a. Lines are fits. Experiments are done in triplicate.

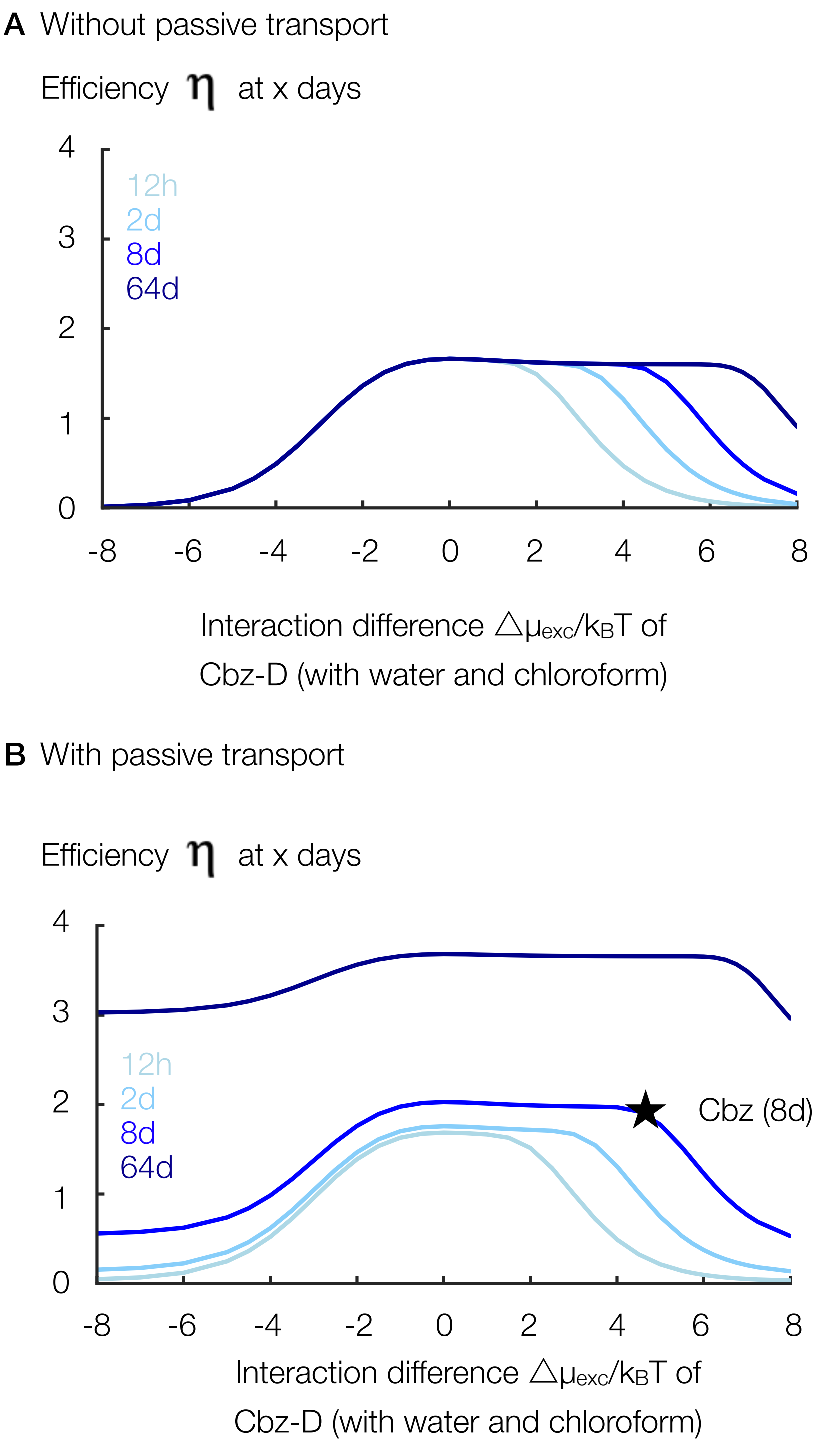

Figure S17. Efficiency at various times as a function of the interaction difference of Cbz-An. (A) without passive transport of precursor, $\boldsymbol{\Delta\mu_{Cbz}} \rightarrow -\infty$. (B) with passive transport of precursor, $\boldsymbol{\Delta\mu_{Cbz}}$ = -11.85. The star indicates the reaction-diffusion model result for Cbz-D as precursor molecule.

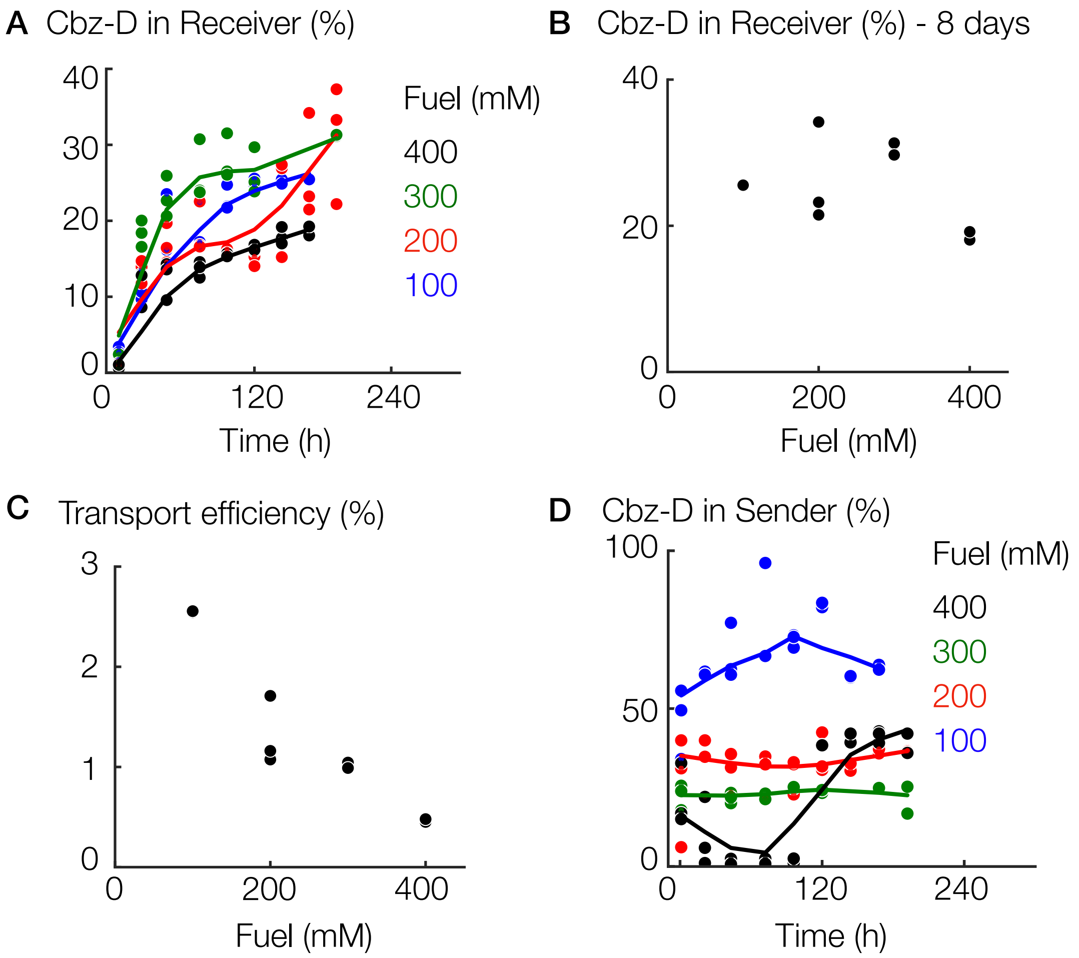

Figure S18. Influence of EDC concentration on the active transport. (A, D) 10 mM Cbz-D fueled with 100/200/300/400 mM EDC (0.35cm sender and receiver phase, 2.54 cm chloroform phase and 1000 rpm) **(A)** Receiver side, **(D)** Sender side. **(B)** Yield (%) of transported Cbz-D after 8 days on the receiver side. **(C)** Transport efficiency as a function of fuel concentration. Based on the amount of Cbz-D transported, we calculated the transport efficiency using ($[Cbz\text{-}D]_{receiver}(t=8d)/[fuel]_{sender}(t=0)$). (**A, D**) Lines are guides for the eyes. The percentage refers to the starting concentration of acid. Experiments were performed in triplicate.

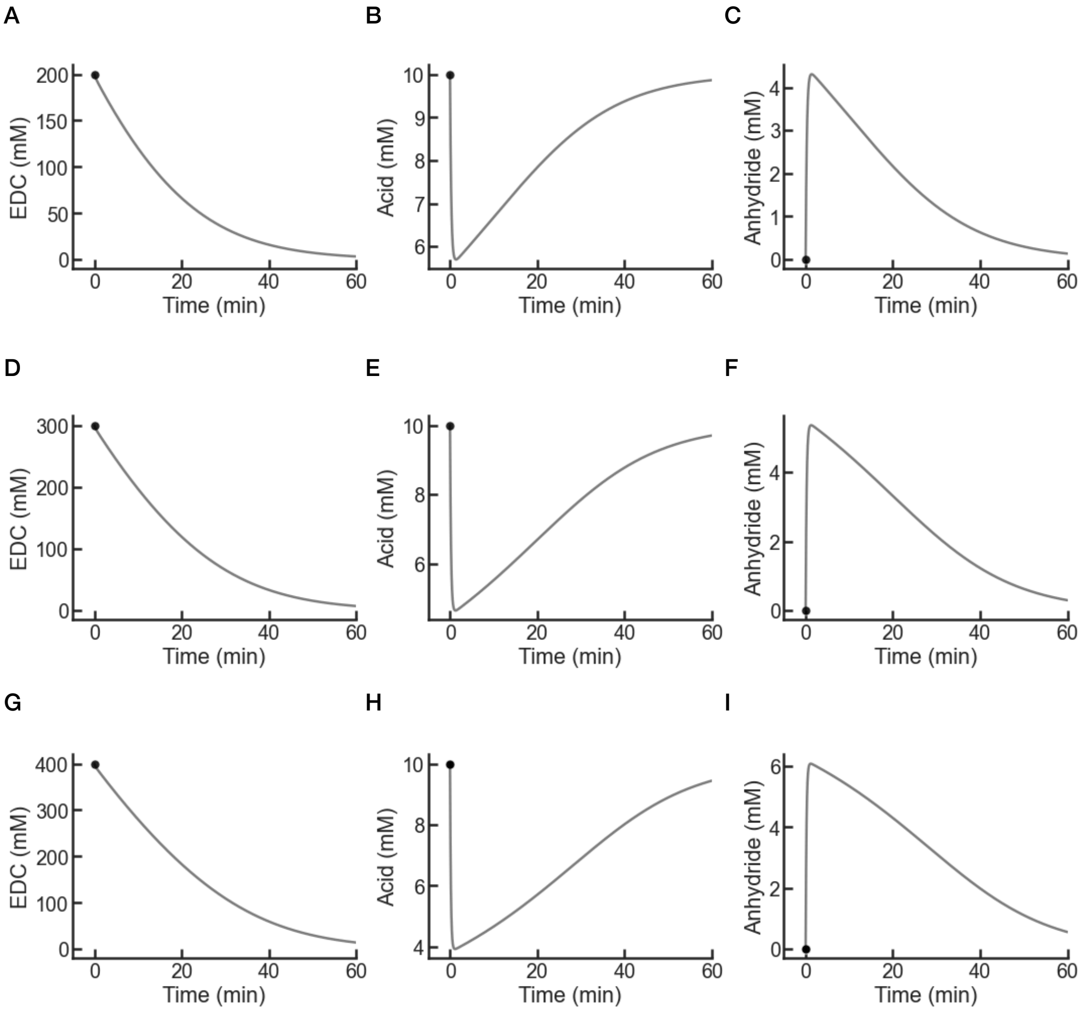

Figure S19. Predicted concentration profiles of 10 mM Cbz-D fueled with 200/300/400 mM EDC. (A, D, G) EDC profiles of. **(B, E, H)** Cbz-D profile, **(C, F, I)** Cbz-anhydride profile. **(A-C)** 200 mM EDC, **(D-F)** 300 mM EDC, **(G-I)** 400 mM EDC. The simulation uses the rate constants obtained by least-square fitting of the set of ODEs that exclude k_a. Lines are fits. The data presented in this figure contains only simulation outputs.

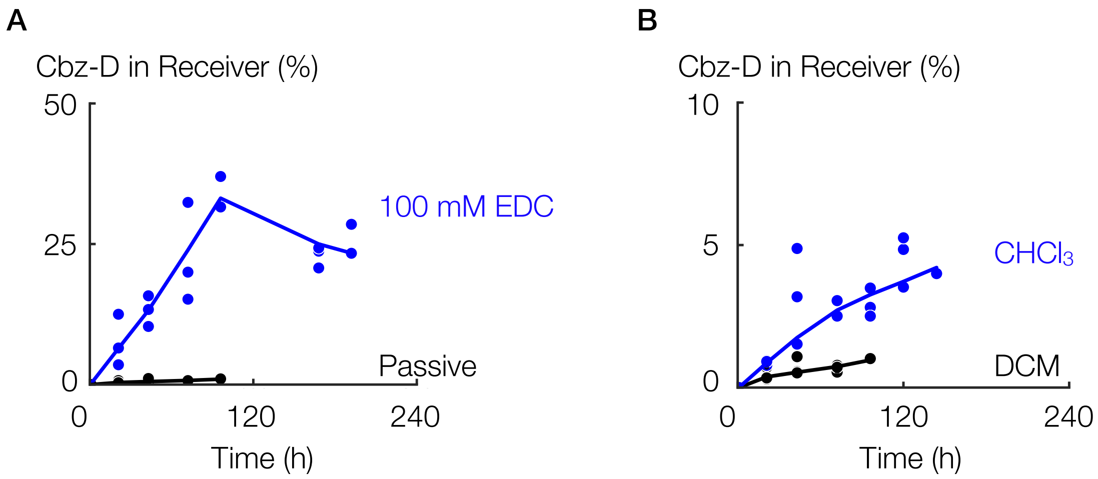

Figure S20. Cbz-D transport profiles of (A) active and passive transport across the hydrophobic domain dichloromethane. **(B)** passive transport across the hydrophobic domain dichlormethane (DCM) and chloroform ($CHCl_3$). Lines are guides for the eye. Experiments were performed in triplicate. The percentage refers to the starting concentration of acid.

SUPPORTING TABLES

Table S1. Characterization of precursors and products.

Name	Structure	Exact mass ($gmol^{-1}$)	Mass found ($gmol^{-1}$)	Retention time (min)	Calibration value ($m.a.u.mM^{-1}$)
**EDC**	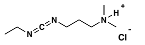	191.12	N/a	5.36	4.04 (220 nm)
**EDU**	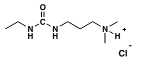	209.13	N/a	N/a	N/a
**Cbz-D**	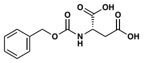	267.24	$[M-H]^+$= 267.93<br>$[M-H]^-$ = 266.73	9.63	0.1686 (254 nm)
**Sulfo phthalic acid**	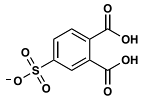	246.19	$[M-H]^-$ = 245.54	3.8	1.504 (254 nm)
**Nitro phthalic acid**	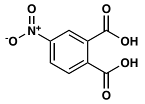	211.01	$[M-H]^-$ = 210.12	8.26	7.46 (254 nm)
**Phthalic acid**	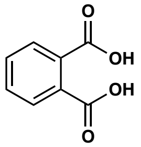	166.03	$[M-H]^-$ = 165.01	8.22	0.849 (254 nm)
**Methoxyphthalic acid**	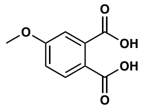	196.04	$[M+H]^+$ = 196.76	8.86	13.45 (254 nm)
**1-pentylamine**	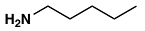	87.1	N/a	7.06	N/a
**Cbz-pentylamide**	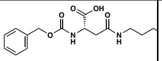<br>Two isomers but only one shown	336.39	$[M-H]^+$= 337.26<br>$[M-H]^-$= 335.79	11.92	0.1686 (254 nm)
**Sulfo phthalic pentyla-mide**	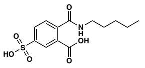<br>Two dimers but only one shown	315.34	$[M-H]^-$ = 314.51	7.34	1.504 (254 nm)
**Nitro phthalic pentyla-mide**	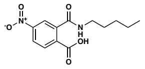<br>Two isomers but only one shown	280.28	$[M-H]^+$= 271.01<br>$[M-H]^-$ = 279.68	10.42	7.46 (254 nm)
**phthalic pentylamide**	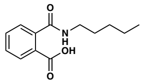<br>Two isomers but only one shown	235.12	$[M+H]^+$ = 235.97	10.635	0.849 (254 nm)
**Methoxy phthalic pen-tylamide**	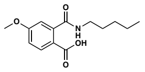<br>Two isomers but only one shown	265.13	$[M+H]^+$ = 265.93	11.17	13.45 (254 nm)

**1-(3-(dimethyla-mino)propyl)-3-ethyl-2-pentylguanidine**	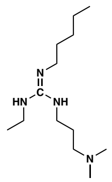	242.41	$[M\text{-}H]^+$= 243.20	8.25	0.198 (254 nm)

Table S2. Rate constant used for least-square fitting with succinic acid derivatives at pH 6 fueled with EDC

	**k_0 (min^{-1})**	**k_1 ($mM.min^{-1}$)**	**k_a**	**$k_4 = k_{hyd}$ (min^{-1})**	**Half-life* (min)**
**Cbz-D**	0.00042	0.00812	0.00000099	2.0	0.347
**Nitro phthalic acid**	0.00042	0.0064	0.000001	5.59	0.124
**Sulfo phtalic acid**	0.00042	0.0203	0.000001	6.95	0.1
**Phthalic acid**	0.00042	0.00884	0.000001	1	0.69
**Methoxyphthalic acid**	0.00042	0.0850	0.000001	0.95	0.72

* Half-life of anhydride, calculated by $\ln(2)/k_4$.

Table S3. Rate constant used for least-square fitting with succinic acid derivatives at pH 6 fueled with EDC

	**k_0 (min^{-1})**	**k_{fuel} ($mM.min^{-1}$)**	**$k_4 = k_{hyd}$ (min^{-1})**	**Half-life* (min)**
**Cbz-D**	0.00042	0.00815	2.05	0.338
**Nitro phthalic acid**	0.00042	0.0068	5.64	0.122
**Sulfo phtalic acid**	0.00042	0.0223	6.91	0.1
**Phthalic acid**	0.00042	0.00984	0.99	0.7
**Methoxyphthalic acid**	0.00042	0.0750	0.92	0.75

* Half-life of anhydride, calculated by $\ln(2)/k_{hyd}$.

Table S4. Anhydride hydrolysis in water-saturated chloroform

	$k_{hyd,CHCl3}$ **(min^{-1})**
**Cbz-D-ahydride**	0.0000048
**Phthalic anhydride**	0.0000031

Table S5. Fit parameters obtained within the reaction-diffusion model.

	$D(\mathrm{cm^2 s^{-1}})$	$r(cms^{-1})$	$\Delta\mu_{ac}(k_B T)$	$\Delta\mu_{an}(k_B T)$
**Cbz-D**	$(3.1 \pm 0.7) \cdot 10^{-4}$	0.50 ± 0.03	-11.86 ± 0.11	4.18 ± 0.18
**Sulfo-phthalic acid**	$(7 \pm 4) \cdot 10^{-3}$	$(8.6 \pm 0.7) \cdot 10^{-3}$	-100	3.0 ± 0.3
**Nitro-phthalic acid**	$(1.8 \pm 0.2) \cdot 10^{-3}$	0.26 ± 0.03	-12.01 ± 0.09	4.4 ± 0.4
**Phthalic a-cid**	$(9 \pm 2) \cdot 10^{-3}$	0.130 ± 0.005	-10.23 ± 0.02	7.00 ± 0.05
**Metoxy-phthalic acid**	$(1.5 \pm 1.4) \cdot 10^{-3}$	0.228 ± 0.03	-14.23 ± 0.14	5.4 ± 0.8

References

[1] Schnitter, F.; Boekhoven, J., A Method to Quench Carbodiimide-Fueled Self-Assembly. *ChemSystemsChem* **2021,** *3* (1), e2000037.

[2] Virtanen, P.; Gommers, R.; Oliphant, T. E.; Haberland, M.; Reddy, T.; Cournapeau, D.; Burovski, E.; Peterson, P.; Weckesser, W.; Bright, J.; van der Walt, S. J.; Brett, M.; Wilson, J.; Millman, K. J.; Mayorov, N.; Nelson, A. R. J. Jones, E.; Kern, R.; Larson, E.; Carey, C. J.; Polat, İ.; Feng, Y.; Moore, E. W.; VanderPlas, J.; Laxalde, D.; Perktold, J.; Cimrman, R.; Henriksen, I.; Quintero, E. A.; Harris, C. R.; Archibald, A. M.; Ribeiro, A. H.; Pedregosa, F.; van Mulbregt, P.; Vijaykumar, A.; Bardelli, A. P.; Rothberg, A.; Hilboll, A.; Kloeckner, A.; Scopatz, A.; Lee, A.; Rokem, A.; Woods, C. N.; Fulton, C.; Masson, C.; Häggström, C.; Fitzgerald, C.; Nicholson, D. A.; Hagen, D. R.; Pasechnik, D. V.; Olivetti, E.; Martin, E.; Wieser, E.; Silva, F.; Lenders, F.; Wilhelm, F.; Young, G.; Price, G. A.; Ingold, G.-L.; Allen, G. E.; Lee, G. R.; Audren, H.; Probst, I.; Dietrich, J. P.; Silterra, J.; Webber, J. T.; Slavič, J.; Nothman, J.; Buchner, J.; Kulick, J.; Schönberger, J. L.; de Miranda Cardoso, J. V.; Reimer, J.; Harrington, J.; Rodríguez, J. L. C.; Nunez-Iglesias, J.; Kuczynski, J.; Tritz, K.; Thoma, M.; Newville, M.; Kümmerer, M.; Bolingbroke, M.; Tartre, M.; Pak, M.; Smith, N. J.; Nowaczyk, N.; Shebanov, N.; Pavlyk, O.; Brodtkorb, P. A.; Lee, P.; McGibbon, R. T.; Feldbauer, R.; Lewis, S.; Tygier, S.; Sievert, S.; Vigna, S.; Peterson, S.; More, S.; Pudlik, T.; Oshima, T.; Pingel, T. J.; Robitaille, T. P.; Spura, T.; Jones, T. R.; Cera, T.; Leslie, T.; Zito, T.; Krauss, T.; Upadhyay, U.; Halchenko, Y. O.; Vázquez-Baeza, Y.; SciPy, C., SciPy 1.0: fundamental algorithms for scientific computing in Python. *Nat. Methods* **2020,** *17* (3), 261-272.

[3] Vallat, R., Pingouin: statistics in Python. *JOSS* **2018,** *3* (31).

[4] X. Chen, H. Soria-Carrera, O. Zozulia, J. Boekhoven, *Chem. Sci.* **2023**, *14*, 12653-12660

[5] L. S. Kariyawasam, C. S. Hartley, *J. Am. Chem. Soc.* **2017**, *139*, 11949-11955.

[6] a) M. Tena-Solsona, B. Rieß, R. K. Grötsch, F. C. Löhrer, C. Wanzke, B. Käsdorf, A. R. Bausch, P. Müller-Buschbaum, O. Lieleg, J. Boekhoven, *Nature Communications* **2017**, *8*, 15895; b) T. Iwasawa, P. Wash, C. Gibson, J. Rebek, Jr., *Tetrahedron* **2007**, *63*, 6506-6511; c) X. Chen, B. A. K. Kriebisch, A. M. Bergmann, J. Boekhoven, *Chem. Sci.* **2023**, *14*, 10176-10183. d) Kriebisch, B. A. K.; Kriebisch, C. M. E.; Bergmann, A. M.; Wanzke, C.; Tena-Solsona, M.; Boekhoven, J., Tuning the Kinetic Trapping in Chemically Fueled Self-Assembly**. *ChemSystemsChem* **2023,** *5* (1), e202200035.

[7] J. Bauermann, S. Laha, P.M. McCall, F. Jülicher, C.A. Weber, *J. Am. Chem. Soc.* **2022,** 144 (42), 19294–19304.

[8] D. Zwicker, *Current Opinion in Colloid & Interface Science* 61 (2022): 101606.
